# Atomically Dispersed Ni‐N‐C Catalysts for Electrochemical CO_2_ Reduction

**DOI:** 10.1002/smll.202412162

**Published:** 2025-01-16

**Authors:** John C. Weiss, Yanghua He, David A. Cullen, Angelica Benavidez, Jeremy D. Jernigen, Hanguang Zhang, Luigi Osmieri, Piotr Zelenay

**Affiliations:** ^1^ Materials Physics and Applications Division Los Alamos National Laboratory Los Alamos NM 87545 USA; ^2^ Center for Nanophase Materials Science Oak Ridge National Laboratory Oak Ridge TN 37830 USA; ^3^ Department of Chemical and Biological Engineering University of New Mexico Albuquerque NM 87131 USA

**Keywords:** carbon dioxide valorization, electron microscopy, heterogeneous electrocatalysis, Ni‐N‐C

## Abstract

The atomic dispersion of nickel in Ni‐N‐C catalysts is key for the selective generation of carbon monoxide through the electrochemical carbon dioxide reduction reaction (CO_2_RR). Herein, the study reports a highly selective, atomically dispersed Ni_1.0%_‐N‐C catalyst with reduced Ni loading compared to previous reports. Extensive materials characterization fails to detect Ni crystalline phases, reveals the highest concentration of atomically dispersed Ni metal, and confirms the presence of the proposed Ni‐N_x_ active site at this reduced loading. The catalyst shows excellent activity and selectivity toward CO generation, with a faradaic efficiency for CO generation (FE_CO_) of 97% and partial current density for CO (j_co_) of ‐9.0 mA cm^−2^ at ‐0.9 V in an electrochemical H‐type cell. CO_2_RR activity and selectivity are also studied by rotating disk electrode (RDE) measurements where transport limitations can be suppressed. It is expected that the utility of these Ni‐N‐C catalysts will lie with tandem CO_2_RR reaction schemes to multi‐carbon (C_2+_) products.

## Graphical abstract

Ni‐N‐C catalysts are effective for electrochemical CO_2_ reduction to CO, but they suffer from the competing hydrogen evolution reaction. A Ni content of 1 wt.% in the precursor produces an atomically dispersed Ni catalyst which achieves 97% faradaic efficiency toward CO. Atomic resolution microscopy confirms atomic dispersion and reveals the formation of Ni particles with higher Ni contents.

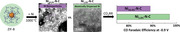

## 1 Introduction

The electrochemical valorization of CO_2_ has surfaced as an intuitive approach to mitigate the greenhouse effects of rising CO_2_ concentrations in the atmosphere.^[^ ^1^, ^2^, ^3^, ^4^ ^]^ During valorization, captured CO_2_ undergoes electrochemical reduction to generate value‐added products (e.g., CO, HCOOH, CH_4_, C_2_H_4_, CH_3_OH, C_2_H_5_OH, etc.), which can be used as fuels and key feedstocks to the chemical industry.^[^ ^5^, ^6^, ^7^ ^]^ As these chemicals are traditionally produced from fossil fuel sources, electrochemical CO_2_ valorization serves as an effective method of reducing fossil fuel reliance and closing the harmful cycle of carbon emissions.

Following the trends of most electrochemical reaction schemes, the electrochemical carbon dioxide reduction reaction (CO_2_RR) has achieved most of its success through metal nanoparticle catalysts.^[^ ^8^, ^9^, ^10^ ^]^ Au‐based catalysts, for instance, have been largely responsible for the high selectivity and partial current density toward CO generation that have been reported in the literature.^[^ ^11^, ^12^, ^13^, ^14^ ^]^ However, Au is a precious metal, and therefore not cost‐effective. Cu‐based catalysts have garnered the most attention for electrochemical CO_2_RR due to their ability to generate more reduced products (CH_4_, C_2_H_4_, C_2_H_5_OH). However, these catalysts struggle to achieve high selectivity, resulting in the need for costly separation steps. Herein lies the appeal of transition metal‐nitrogen‐carbon (M‐N‐C where M = Fe, Co, Ni, Mn, Cu, etc.) electrocatalysts. This class of materials relies on atomically dispersed M‐N_x_ species to catalyze a number of important electrochemical reactions, including ORR, N_2_RR, and CO_2_RR.^[^ ^15^, ^16^, ^17^, ^18^, ^19^, ^20^, ^21^, ^22^ ^]^ Fe‐N‐C and Co‐N‐C catalysts began to gain traction in the late 2000s due to their high initial ORR activity and significantly reduced cost relative to platinum group metal (PGM)‐based benchmarks.^[^ ^23^, ^24^, ^25^ ^]^ However, M‐N‐C materials have struggled to advance past early stages of development due to acute performance degradation in acidic media.^[^ ^26^, ^27^, ^28^, ^29^ ^]^ Here lies another appeal of using M‐N‐C catalysts for CO_2_RR, which is typically catalyzed in near‐neutral pH to maximize the generation of high‐value hydrocarbon products while limiting both the formation of carbonate and the undesired HER.^[^ ^30^, ^31^ ^]^

Here, we have aimed to optimize Ni‐N‐C electrocatalysts for CO_2_RR, which have shown promise for the generation of CO at high selectivity, faradaic efficiency (FE) > 90%, and moderate current densities (∼10 mA cm^−2^) in a three‐electrode H‐type electrochemical cell (H‐cell).^[^ ^32^, ^33^, ^34^, ^35^ ^]^ Several groups have proposed that the electroreduction of CO_2_ to CO occurs at single‐atom Ni sites coordinated by N atoms (Ni‐N_x_) in high surface area carbon domains.^[^ ^32^, ^35^, ^36^ ^]^ The synthesis of these structures is often achieved through the pyrolysis of Ni precursors together with a zeolitic imidazolate framework (ZIF‐8), which in turn is converted to a nitrogen‐doped carbon host for Ni‐N_x_. As Ni precursors tend to be reduced into metallic Ni particles during pyrolysis, the selectivity of these catalysts is often limited by the competing HER.^[^ ^37^, ^38^, ^39^ ^]^ This sintering phenomenon is well‐documented in the synthesis of M‐N‐C catalysts for a variety of reactions and has been solved by tuning the metal loading to 1 wt.%, or less, in the precursors.^[^ ^40^ ^]^ In surveying the current literature reports on atomically dispersed Ni‐N‐C electrocatalysts for CO_2_RR, we have pinpointed several points for improvement:

i) Unusually high Ni contents in the precursor, which can lead to the formation of Ni nanoparticles in Ni‐N‐C catalysts. Such species are HER active.
ii) The presence of carbon nanotubes in microscopy imaging, which typically manifests as a result of nanoparticle growth during the pyrolysis of MOF‐derived catalysts.
iii) The lack of correlation between Ni loading and CO_2_RR selectivity toward CO (i.e., a steady decrease in CO generation with more numerous and/or larger Ni nanoparticles at higher Ni loadings).

Therefore, we have sought to further investigate Ni‐N‐C for CO_2_RR to CO through modifying a previously reported ZIF‐8 synthesis method^[^ ^32^ ^]^ with reduced Ni loadings. Through extensive annular dark‐field scanning transmission electron microscopy (ADF‐STEM) studies in parallel with energy‐dispersive X‐ray spectroscopy (EDS) and electron energy loss spectroscopy (EELS), the dispersion of Ni metal in these layers has been thoroughly characterized. Electrochemical measurements coupled with gas chromatography (GC) yielded a distinct trend relating Ni loading to CO_2_RR performance and selectivity, along with an optimized, atomically dispersed catalyst (Ni_1.0%_‐N‐C) with a selectivity for CO (FE_CO_) of 97% at ‐0.9 V. Additional electrochemical testing was performed using a rotating disk electrode (RDE) to isolate the CO_2_RR kinetics at the Ni‐N‐C catalysts by limiting the accumulation of gas products at the surface of the working electrode. As a result, we not only acknowledge the success of these catalysts for CO and syngas production, but also hope to explore their utility in tandem (or cascade) systems for the generation of C_2+_ products.

## 2 Experimental Section

### 2.1 Materials

Zn(NO_3_)_2_·6H_2_O, Ni(NO_3_)2·6H_2_O, 2‐methylimidazole, KHCO_3_, ethanol, methanol, and n‐hexane were purchased from Sigma Aldrich and used as received. 5 wt% Nafion dispersion (D521) was purchased from Fuel Cell Store. Ultrapure deionized water (DI) was obtained with a Millipore Milli‐Q system (resistivity: 18.2 MΩ cm).

### 2.2 Synthesis of ZIF‐8

11.16 g of Zn(NO_3_)_2_·6H_2_O and 12.32 g of 2‐methylimidazole were separately dissolved in 100 mL of methanol. The solutions were then mixed in a 500 mL round bottom flask and stirred at 40 °C for 8 hours under inert conditions to form a white precipitate. This precipitate was then centrifuged at 9000 rpm and triple‐washed with ethanol before being dried under vacuum at 65 °C for 4 hours to obtain a white ZIF‐8 powder.

### 2.3 Synthesis of Ni‐N‐C Catalysts

0.25 g of ground ZIF‐8 powder was transferred into a small glass vial and dispersed in 10 mL of n‐hexane under constant mixing. The vial was capped to prevent the evaporation of n‐hexane. In a separate vial, 1.00 g of Ni(NO_3_)_2_·6H_2_O was dissolved in 10 mL of deionized water. Specific volumes of this Ni(NO_3_)_2_·6H_2_O solution were then added to the ZIF‐8 dispersion with respect to the atomic ratio of Ni‐to‐Zn in the precursor (e.g., 131 µL for Ni_1.0%_‐N‐C), which are shown in **Table** **1**. The resulting precursor dispersion was then stirred for 2 hours until obtainment of a homogeneous green paste and then filtered under vacuum. Next, the paste was collected and dried under vacuum at 65 °C for 4 hours to obtain a powder. After being ground with a mortar and pestle, the Ni‐doped ZIF‐8 powder was pyrolyzed at 1000 °C under Ar flow for 2 hours with a 1‐hour ramp‐up time to obtain the Ni‐N‐C electrocatalysts.

**Table 1:** Ni‐N‐C catalysts from this work expressed in terms of Ni wt.%, and their respective Ni:Zn atomic ratios in the precursor.

Catalyst	Ni:Zn atomic ratio
Ni_0.0%_‐N‐C	Ni‐free
Ni_0.5%_‐N‐C	1:20
Ni_1.0%_‐N‐C	1:10
Ni_1.9%_‐N‐C	1:5
Ni_4.2%_‐N‐C	1:2
Ni_6.9%_‐N‐C	1:1
Ni_10.3%_‐N‐C	2:1

### 2.4 Materials Characterization

The presence of crystalline species was screened with X‐ray diffraction (XRD). Measurements were performed with a Siemens D5000 diffractometer using a Cu K_α_ radiation source and a graphite monochromator. XRD diffractograms were measured in the 2θ range between 20° and 80°, with a 0.1° step. Jade9 software was used to determine the crystallographic phases associated with the peaks in the XRD diffractograms. XPS measurements were performed on a Kratos Ultra DLD spectrometer using a monochromatic Al K_α_ source operating at 150 W (1486.6 eV). The operating pressure was 2 10^−9^ Torr. High‐resolution spectra were acquired at a pass energy of 20 eV. XPS data was processed using Casa XPS software. Morphology and atomic dispersion of metals were investigated through annular dark‐field scanning transmission electron microscopy (ADF‐STEM) in parallel with electron energy loss spectroscopy (EELS) and energy‐dispersive X‐ray spectroscopy (EDS). The ADF‐STEM images were recorded on a probe‐corrected JEOL NEOARM operated at 80 kV and equipped with a cold field emission gun.

### 2.5 Electrochemical Measurements and Product Analysis

5 mg of ground Ni‐N‐C powder was dispersed in 1 mL of an isopropanol/DI water mixture (3:1 vol) and 50 µL of 5% Nafion suspension in alcohols. The ink was sonicated for 20 minutes before being drop‐cast on the working electrode support at a fixed loading of 0.15 mg cm^−2^. All electrochemical measurements were performed at ambient conditions.

The electrocatalytic activity was first measured in a three‐electrode cell configuration using a rotating disk electrode (RDE) setup. A 0.247 cm^2^ glassy carbon disk electrode loaded with Ni‐N‐C at 0.15 mg cm^−2^ was used as the working electrode along with a saturated Ag/AgCl reference electrode and a Pt‐wire counter electrode. All electrodes were submersed in 0.1 M KHCO_3_ saturated with either ultra‐high purity grade Ar (≥99.999%) or CO_2_ (≥99.99%) in a single‐compartment, 5‐neck glass cell. All potentials are reported versus the reversible hydrogen electrode (RHE). RDE measurements were conducted in the following order:

i) *Break‐in in Ar‐saturated 0.1 M KHCO_3_*. Cyclic voltammetry (CV): 10 cycles from 0.0 to ‐1.0 V at 20 mV s^−1^ scan rate and 1600 rpm RDE rotation speed.
ii) *1^st^ activity measurement in Ar‐saturated 0.1 M KHCO_3_*. Staircase voltammetry (SV): one scan from 0.0 to ‐1.0 Vin 25 mV potential‐step increments, 20 s hold time per step, varied rotation speed (100, 400, 900, 1600, and 2500 rpm).
iii) *Break‐in in CO_2_‐saturated 0.1 M KHCO_3_*. CV: 10 cycles from 0.0 to ‐1.0 V at 20 mV s^−1^ scan rate and 1600 rpm RDE rotation speed.
iv) *2^nd^ activity measurement in CO_2_‐saturated 0.1 M KHCO_3_*. SV: one scan from 0.0 to ‐1.0 V in 25 mV potential‐step increments, 20 s hold time per step, varied rotation speed (100, 400, 900, 1600, and 2500 rpm).

Electrochemical measurements were also performed in a three‐electrode H‐cell, with two compartments separated by a proton‐exchange membrane (Nafion N115). Working electrodes were fabricated on a carbon paper (Sigracet 29AA), with a total area of 1.0 cm^2^. Each H‐cell compartment was filled with 30 mL of 0.1 M KHCO_3_. A Pt wire was used as the counter electrode and submersed in the anolyte, while the working electrode and a saturated Ag/AgCl reference electrode were submersed in the catholyte. Ultra‐high purity grade CO_2_ (≥99.99%) was fed at 20 sccm to the catholyte using a mass flow controller. Gas products were analyzed downstream from the H‐cell using an Agilent 990 Micro Gas Chromatograph (GC) equipped with both Molesieve 10A and CP‐PoraPlot Q columns and a dual‐channel thermal conductivity detector (TCD). H‐cell voltammetry experiments were performed in the following order:

i) *Break‐in*. CV: Ten cycles from 0.0 to ‐1.0 V at 20 mV s^−1^.
ii) *Activity measurement*. SV: one reverse scan from 0.0 to ‐1.0 V in 25 mV potential‐step increments and 20 s hold time per step.
iii) *Potential hold experiment*. Chronoamperometry (CA): varied potentials (‐0.7, ‐0.8, ‐0.9, and ‐1.0 V) using 30‐minute hold time, current measurements acquired every 10 s. Downstream GC for gas product analysis.

## 3 Results and Discussion

### 3.1 Physicochemical Characterization

**Figure 1:**
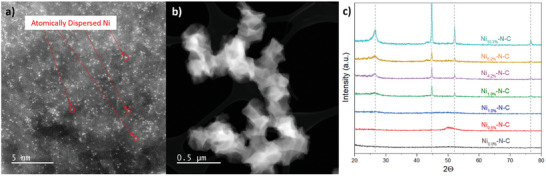
ADF‐STEM images showing a) the atomic dispersion of Ni in Ni_1.0%_‐N‐C, and b) Ni_1.0%_‐N‐C nanoparticles post‐pyrolysis and their resemblance to dodecahedral ZIF‐8. c) XRD patterns for all Ni‐N‐C catalysts, showing a lack of Ni crystalline species in Ni_1.0%_‐N‐C, Ni_0.5%_‐N‐C, Ni_0.0%_‐N‐C.

Ni‐N‐C was synthesized by a typical ZIF‐8‐derived method. In this procedure, ZIF‐8 is first synthesized, then doped with active Ni species, and finally pyrolyzed at 1000 °C under inert atmosphere. Pyrolysis is necessary for the formation of atomically dispersed Ni‐N_x_ CO_2_RR active sites (**Figure** **1**a) and removal of the spectator Zn species (by evaporation), while the rhombic dodecahedral ZIF‐8 geometry is somewhat maintained (Figure 1b). However, pyrolysis also promotes the formation of undesirable Ni nanoparticles once excessive Ni content (>1wt%) is used in the precursor.

To study the formation of crystalline Ni phases, XRD patterns of each Ni‐N‐C electrocatalyst were obtained between 20° and 80° (Figure 1c). Peaks corresponding to the Ni (111), Ni (200), and Ni (220) planes of metallic Ni (PDF # 00‐004‐0850) appeared at 44°, 51°, and 76°, respectively, for molar ratios of 1:5 [Ni‐to‐Zn] and higher. These crystalline Ni‐containing species provide evidence for the formation of Ni nanoparticles. Also, the peak at 26°, which corresponds to graphitic C (002), became sharp for Ni loadings greater than 1.0 wt%. As well‐documented in previous works,^[^ ^41^, ^42^, ^43^, ^44^ ^]^ the graphitization of carbon domains is often catalyzed by metal nanoparticles, and therefore serves as a key indicator to the formation of Ni nanoparticles herein.

ADF‐STEM provided direct evidence for the generation of atomically dispersed Ni species in Ni‐N‐C catalysts. Figure 1a displays a region rich in atomically dispersed Ni found in a Ni‐N‐C catalyst derived from precursor containing 1.0 wt% Ni. EELS was coupled with ADF‐STEM to confirm the identity of the atomically dispersed metal and detect any potential coordination by nearby species (Figures S1–S5, Supporting Information). Peaks found at 855 eV, 405 eV, and 290 eV confirmed the identity of the metal as Ni and the coordinating species as N and C, respectively, for these regions in Ni_0.5%_‐N‐C, Ni_1.0%_‐N‐C, Ni_1.9%_‐N‐C, Ni_4.2%_‐N‐C, and Ni_6.9%_‐N‐C. We therefore have demonstrated the presence of the Ni‐N_x_ species, assumed to be the primary active site for CO_2_RR.

**Figure 2:**
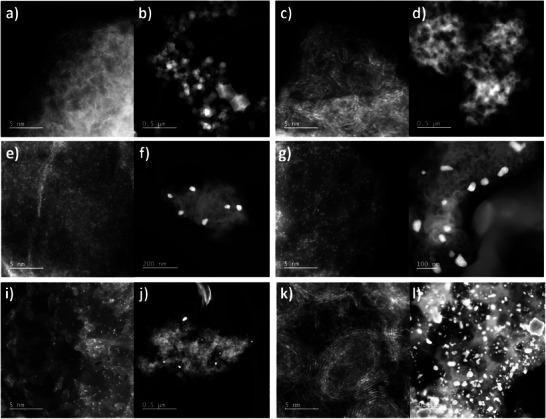
ADF‐STEM images of a,b) Ni_0.0%_‐N‐C, c,d) Ni_0.5%_‐N‐C, e,f) Ni_1.9%_‐N‐C, g,h) Ni_4.2%_‐N‐C, i,j) Ni_6.9%_‐N‐C, and k,l) Ni_10.3%_‐N‐C.

**Figure** **2** provides comprehensive AFD‐STEM imaging of all other Ni‐N‐C catalysts synthesized herein. No metal nanoparticle regions were detected in both Ni_0.0%_‐N‐C and Ni_0.5%_‐N‐C (Figure 2a–d), while a resemblance to the dodecahedral geometry of ZIF‐8 was found (Figure 2b,d). In the case of Ni_0.0%_‐N‐C, a small number of atomically dispersed species were detected in Figure 2a. This is attributed to the presence of leftover Zn atoms from the ZIF‐8 precursor, the entirety of which were not evaporated out during pyrolysis. While atomically dispersed regions were found in Ni_0.5%_‐N‐C, Ni_1.9%_‐N‐C, Ni_4.2%_‐N‐C, and Ni_6.9%_‐N‐C alike (Figure 2c,e,g,i), nanoparticles regions were also found in Ni_1.9%_‐N‐C, Ni_4.2%_‐N‐C, and Ni_6.9%_‐N‐C along with the complete destruction of the ZIF‐8 geometry (Figure 2f,h). In the case of Ni_10.3%_‐N‐C, excessive Ni loading in the precursor resulted in no regions with detectable atomically dispersed sites through ADF‐STEM imaging (Figure 2k,l). Instead, more ordered carbon domains were found to house Ni agglomerates embedded in graphitized carbon species, such as the nano‐onions found in Figure 2k.

**Table** **2** reports the relative composition of relevant atoms in the atomically dispersed regions of Ni‐N‐C, as probed by EDS (Figures S6–S12, Supporting Information). Each value represents an average of three atomically dispersed regions. No compositions are reported for both Ni_0.0%_‐N‐C and Ni_10.3%_‐N‐C due to the lack of atomically dispersed regions. Notably, the atomically dispersed regions in Ni_1.0%_‐N‐C were the richest in Ni, with the formation of Ni nanoparticles at higher Ni loadings resulting in a steady decrease in the content of atomically dispersed Ni species. Zn composition was also probed by EDS, as Zn species from the ZIF‐8 precursor are sometimes left unevaporated in Ni‐N‐C catalysts following pyrolysis and may be catalytically active. Higher Zn concentrations were seen in samples void of crystalline Ni phases (Ni_0.5%_‐N‐C and Ni_1.0%_‐N‐C), while all Ni nanoparticle‐containing samples were Zn‐free. This may be attributed to the preservation of ZIF‐8 structures in more atomically dispersed samples, while the migration of Ni atoms to nanoparticles and the breakdown of ZIF‐8 structure can facilitate Zn removal during pyrolysis.

**Table 2:** Normalized atomic percent of elements in atomically dispersed regions of Ni‐N‐C, as determined by EDS.

Catalyst	Ni	N	C	Zn
Ni_0.5%_‐N‐C	0.11%	1.8%	98%	0.21%
Ni_1.0%_‐N‐C	0.47%	2.7%	97%	0.17%
Ni_1.9%_‐N‐C	0.38%	1.7%	98%	0.00%
Ni_4.2%_‐N‐C	0.32%	1.2%	99%	0.00%
Ni_6.9%_‐N‐C	0.24%	1.3%	98%	0.00%

X‐ray photoelectron spectroscopy (XPS) experiments (Figures S13–S16, Supporting Information) were conducted to identify near surface species and their relative compositions (Tables S1–S4, Supporting Information). Survey scans revealed that all samples consisted of Ni, N, O, and C (consistent with EDS results), with Zn (∼1022 eV) detected only in the samples free of crystalline Ni (also confirming the EDS results). While found in all Ni‐N‐C samples, pyridinic N (398.3 eV) was most abundant in Ni_1.0%_‐N‐C. The pyridinic‐N species are generally considered favorable for the formation of active M‐N‐C sites.^[^ ^45^, ^46^, ^47^ ^]^ The content of Ni‐N (399.6 eV) was also high in optimized Ni_1.0%_‐N‐C at 2.46%.

### 3.2 CO_2_RR Performance

CO_2_RR performance (activity and selectivity) of catalysts was evaluated in an H‐cell, which was sealed to allow for the determination of gas product selectivity. The H‐cell represents a relatively simple approach to performance evaluation compared to a full CO_2_ electrolysis cell (i.e., flow cell or zero‐gap cell). Gas chromatography was used downstream from the H‐cell to determine product selectivity. However, an inherent limitation of the H‐cell is the formation of gas bubbles at the working electrode. Thus, to validate the H‐cell results, RDE measurements were performed using the same catalyst loading (0.15 mg cm^−2^) and electrolyte (0.1 M KHCO_3_) as used in H‐cell testing. In RDE experiments, we aimed to isolate the CO_2_RR kinetic behavior at Ni‐N‐C electrocatalysts while predicting selectivity.

The disk rotation speed in RDE experiments varied from 100 to 2500 rpm in CO_2_‐saturated electrolyte to accelerate mass transport of dissolved CO_2_ while minimizing the effect of gas bubble formation on measured current densities (Figure S17, Supporting Information). In Ar‐saturated electrolyte, where CO_2_RR is suppressed and only HER is expected to occur, the current density was practically independent of the rotation speed. However, in CO_2_‐saturated electrolyte, where both CO_2_RR and HER are taking place at the same time, considerable loss in the current density was observed at rotation speeds lower than 400 rpm at potentials below −0.7 V. That loss was caused by the formation of a large gas bubble, assumed to be primarily CO forming and remaining on the working electrode tip. By increasing rotation speed to 1600 rpm and above, it was possible to remove gas bubbles from the electrode surface, which resulted in the convergence of the current density plots recorded at rotation speeds 1600 and 2500 rpm. It was therefore assumed that the system was neither CO_2_ transport‐ nor bubble formation‐limited at 2500 rpm, and this disk rotation speed was used in the follow‐up experiments.

**Figure 3:**
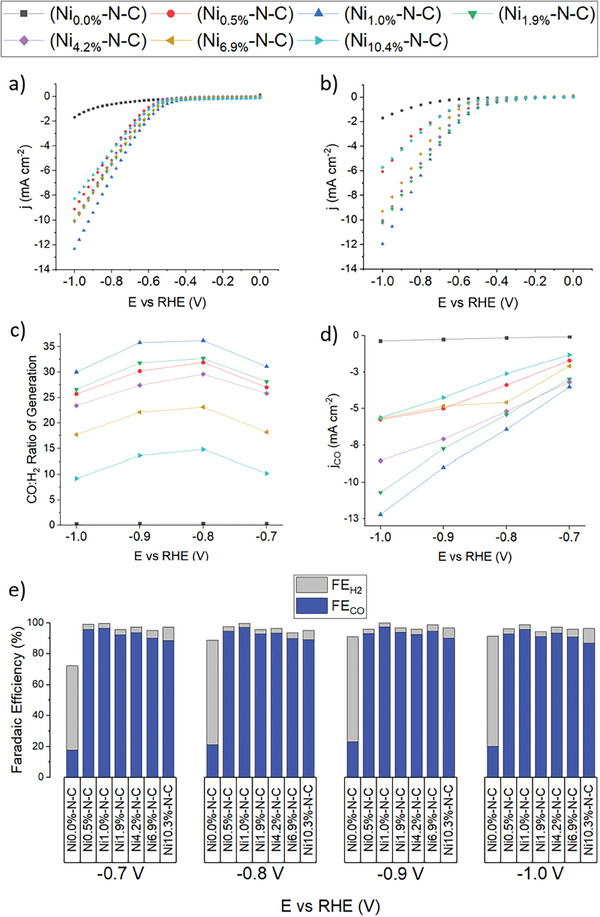
Potential‐dependent CO_2_RR performance of Ni‐N‐C in 0.1 M KHCO_3_. a) Staircase voltammetry obtained by RDE at 2500 rpm. b) Staircase voltammetry obtained by H‐cell. c) Ratio of CO:H_2_ generation by H‐cell. d) Partial current density in H‐cell attributed to CO generation. (e) Faradaic efficiency distribution of CO and H_2_ as a function of potential in H‐cell.

Comparison of the staircase voltammetry (SCV) plots for Ni‐N‐C catalysts in CO_2_‐saturated electrolyte reveals the highest activity of Ni_1.0%_‐N‐C at all observed overpotentials (**Figure** **3**a). This is the only catalyst to achieve a current density of ‐12 mV cm^−2^ at ‐1.0 V, while catalysts synthesized with increasingly higher Ni contents in the precursor demonstrated progressively worse CO_2_RR activity. Consequently, Ni_1.0%_‐N‐C is likely to have the highest surface concentration of Ni‐N_x_ active sites, a result corroborated by the materials characterization data described above.

While gas products could not be directly analyzed in RDE experiments, selectivity was probed by comparing current density measured for each catalyst in CO_2_‐ and Ar‐saturated KHCO_3_ electrolytes (Figure S18, Supporting Information). By determining the difference in current density (Δj) in these two cases at various RDE potentials (Table S5, Supporting Information), selectivity can be qualitatively estimated. In line with the FE results from H‐cell experiments, Ni_1.0%_‐N‐C exhibited the largest Δj at relevant potentials, including Δj = ‐6.2 mA cm^−2^ at ‐0.90 V, while less selective catalysts exhibited reduced Δj. Ni_0.0%_‐N‐C was the only catalyst that showed larger current densities in CO_2_‐free media than CO_2_‐rich media (CO_2_‐saturated 0.1 M KHCO_3_) at high potentials (E ≤ ‐0.825 V). The likely explanation for this observation is that CO_2_ adsorption on Ni_0.0%_‐N‐C in CO_2_‐rich media suppresses its HER activity. A competing surface mechanism of this kind cannot be ruled out for any of the Ni‐containing catalysts either, which implies that Δj cannot be exclusively attributed to the generation of CO only on CO_2_RR active sites. Despite the lack of Ni species in Ni_0.0%_‐N‐C, this catalyst was still shown to generate small amounts of CO. Therefore, the contribution of C, N, and Zn species toward CO_2_RR cannot be ruled out for any of the catalysts reported herein. However, Figure 3a,b show the significantly reduced overall activity of Ni_0.0%_‐N‐C relative to all of the Ni‐containing catalysts, suggesting that the activity of C, N, and Zn species in these catalysts is small relative to Ni.

SCV plots recorded in the H‐cell (Figure 3b) revealed a similar trend to that observed in RDE experiments, with Ni_1.0%_‐N‐C achieving high current densities at CO_2_RR potentials under −0.7 V. Catalysts with higher Ni contents again exhibited lower current densities. However, the current density decrease was more pronounced than in RDE experiments. This dramatic loss is attributed to mass transport limitations caused by the bubbles gradually accumulating on the surface of the H‐cell cathode at high potentials.

Downstream from the H‐cell, CO_2_RR gas products were collected and analyzed by gas chromatography both 10 and 25 minutes into the chronoamperometric experiment to ensure steady state conditions and the product quantities averaged. Ni_1.0%‐_N‐C achieved the highest CO:H_2_ generation ratio of 36:1 at −0.8 V (Figure 3c). Ni‐N‐C catalysts with higher Ni contents again demonstrated smaller FE_CO_ and CO:H_2_ generation ratios. Figure 3d compares partial current density of CO generation (j_CO_) for each catalyst at different potentials. Ni_1.0%_‐N‐C demonstrated the largest j_CO_ of ‐12 mA cm^−2^ at ‐1.0 V. The same catalyst also achieved the highest selectivity for CO of 97.2% at −0.9 V (Figure 3e). The consistently high selectivity for CO measured with the Ni_1.0%_‐N‐C catalyst (FE_CO_ > 95%) across the studied potential range is not only a notable result for CO synthesis and atmospheric CO_2_ removal; it may also be useful for tandem CO_2_RR schemes that target C_2+_ product generation anywhere between ‐0.6 and ‐1.0 V through a CO intermediate.

### 3.3 The Utility of Ni‐N‐C

The Ni_1.0%_‐N‐C catalyst synthesized herein is both active and highly selective toward CO generation. While this result is significant, CO is often less economically desirable than multi‐carbon CO_2_RR products due to its low market value and relative abundance in the chemical industry. The selective generation of further reduced CO_2_RR products, such as ethylene, methane, and short chain alcohols, are even more desirable, leading us to discuss the utility of Ni‐N‐C in the greater landscape of CO_2_ valorization.

**Figure 4:**
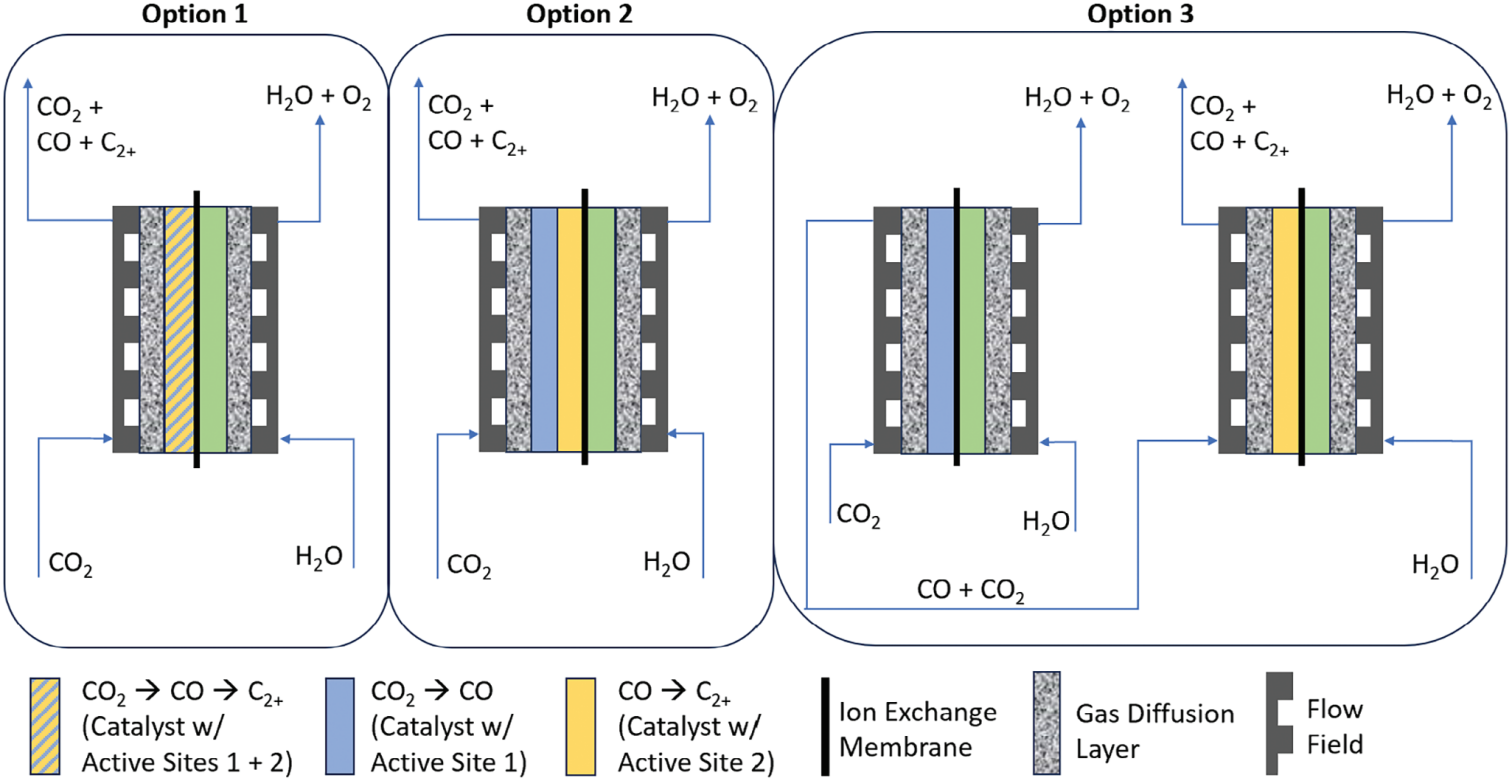
Different proposed tandem (or cascade) methods to achieve C_2+_ product generation in CO_2_ electrolyzers.

The different proposed CO_2_RR reaction pathways are well summarized by Kibria et al.,^[^ ^48^ ^]^ where all multi‐carbon products are thought to be first derived from a *CO intermediate. C‐C dimers are then formed with the help of a second CO species before the subsequent pathways to different products diverge to their completion. For catalysts that target the synthesis of one active site (e.g., Cu nanoparticles), it is therefore difficult to form *CO at one site and selectively achieve C‐C dimerization at another. This brings up the need for multiple active sites in CO_2_ electrolyzer cathodes or multi‐step reaction schemes, as two distinct mechanisms are required to achieve multi‐carbon products.^[^ ^49^ ^]^ Three possible options for tandem generation of multi‐carbon products are schematically depicted in **Figure** **4**. They are as follows:

1. A single catalyst layer with two different active sites: the first to form CO and the second to form C‐C dimers.
2. Two catalyst layers in tandem within the same electrode in a single electrolyzer, each with different active sites: the first one to form CO and provide a mixed reagents feed for the second to form C‐C dimers.
3. Two electrolyzers in tandem, each with a different cathode catalyst: the first one to form CO and provide a mixed reagent feed for the second to form C‐C dimers.

Option 1 was proposed by Wang et al in 2019,^[^ ^50^ ^]^ where separately synthesized Ni‐N‐C and CuO_x_/C nanoparticle catalysts were mixed and tested under varying CO_2_/CO feeds. The most fundamental takeaway from this work was the importance of a mixed CO_2_ + CO feed to improve C_2+_ product selectivity at CuO_x_/C. However, physically mixed Ni‐N‐C and CuO_x_/C catalysts were not selective toward C_2+_ products. This resulted in the development of an improved tandem system by the same authors, which used a dual electrolyzer configuration, more closely resembling Option 3 above.^[^ ^51^ ^]^ Although the introduction of a second electrolyzer, or a thermochemical reactor, inevitably adds cost and complexity to the CO_2_RR scheme, it has been proposed several times in the literature with a few notable successes.^[^ ^51^, ^52^ ^]^ This success has been attributed to the ability to manipulate reaction conditions, including pH, which significantly impacts CO_2_RR product selectivity.

The Ni‐N‐C catalyst is likely to turn over CO_2_ to CO at sufficient rates to assure a mixed CO_2_/CO feed for the second reaction. However, the probability of CO_2_‐CO recombination to C_2+_ products at Cu‐based nanoparticles randomly mixed with Ni‐N‐C within the same catalyst layer is very low. Option 2 therefore presents a favorable, single‐electrolyzer reaction scheme that avoids the design and synthesis of a complex, dual‐active‐site cathode. For this option, the placement of Cu‐based nanoparticles must be downstream from the Ni‐N‐C catalyst in a regular fashion. As depicted in Figure 4, the cathode would therefore be made of alternating layers of the two catalysts, introducing both transport and ionic conductivity resistances.

To realize Option 1 as an effective method of C_2+_ product generation, we hypothesize that a single catalyst layer must be synthesized with NiN_x_ and Cu‐based active sites (or similar), positioned adjacent to one another. This would facilitate the transfer of CO intermediates from NiN_x_ to Cu‐based and thereby boost C_2+_ product selectivity. Such a structure could be achieved through the development or adaptation of a deposition technique, such as strong electrostatic adsorption,^[^ ^53^, ^54^, ^55^, ^56^ ^]^ under conditions that would preserve the NiN_x_ active site. Given the extremely low Ni content (<1 wt.%) and high C content (>95%) in the Ni‐N‐C catalysts presented herein, one could use the Ni‐N‐C catalyst with its high surface area and porosity as a support for the deposition of an additional metal or metal oxide species.

## 4 Conclusion

In this work, we synthesized a family of Ni‐N‐C catalysts for CO_2_RR via the pyrolysis of ZIF‐8 precursors with varying Ni contents. The Ni_1.0%_‐N‐C catalyst with low Ni content of 1 wt% and a Ni‐to‐Zn ratio of 1:10 in the precursor exhibited the best performance for CO generation in terms of both activity and selectivity (FE_CO_ > 97% and j_co_ = ‐9.0 mA cm^−2^ at ‐0.9 V). This performance is attributed to the high density of atomically dispersed Ni species (0.47%) relative to all other synthesized Ni‐N‐C catalysts, as probed by ADF‐STEM imaging coupled with EDS. ADF‐STEM coupled with EELS also suggested the coordination of Ni by N atoms in the domains rich in atomically dispersed sites. Together, these results point to the presence of Ni‐N_x_ species as active sites for CO generation. Unlike Ni‐N‐C catalysts derived from precursors with Ni content greater than 1.0 wt.%, XPS did not reveal presence of any crystalline Ni phases in Ni_1.0%_‐N‐C. It is the presence of Ni nanoparticles that is likely responsible for more substantial HER activity in catalysts with higher Ni content in their precursors, e.g., Ni_10.3%_‐N‐C. Thanks to their activity and selectivity for CO generation, together with the high surface area of the carbon matrix, Ni_1.0%_‐N‐C and other low Ni‐content catalysts in this class are highly promising materials for tandem CO_2_RR electrocatalysis systems for the generation of multi‐carbon compounds.

## Conflict of Interest

The authors declare no conflict of interest.

## Supporting information



Supporting Information

## Data Availability

The data that support the findings of this study are available from the corresponding author upon reasonable request.

## References

[1] J. Wu , Y. Huang , W. Ye , Y. Li , Adv. Sci. 2017, 4, 1700194. 10.1002/advs.201700194 PMC5700640 29201614

[2] X. Li , S. Wang , L. Li , Y. Sun , Y. Xie , J. Am. Chem. Soc. 2020, 142, 9567. 32357008 10.1021/jacs.0c02973

[3] P. Saha , S. Amanullah , A. Dey , Acc. Chem. Res. 2022, 55, 134. 34989553 10.1021/acs.accounts.1c00678

[4] S. Zhang , Q. Fan , R. Xia , T. J. Meyer , Acc. Chem. Res. 2020, 53, 255. 31913013 10.1021/acs.accounts.9b00496

[5] P. Nagarajan , I. J. Augustine , M. B. Ross , Cell Rep. Phys. Sci. 2023, 4, 101472.

[6] Z. Kou , X. Li , T. Wang , Y. Ma , W. Zang , G. Nie , J. Wang , Fundam., Electrochem. Energy Rev. 2022, 5, 82.

[7] C. Chen , J. F. Khosrowabadi Kotyk , S. W. Sheehan , Chem 2018, 4, 2571.

[8] Q. Chen , P. Tsiakaras , P. Shen , Catalysts 2022, 12, 1348.

[9] J. Yu , J. Wang , Y. Ma , J. Zhou , Y. Wang , P. Lu , J. Yin , R. Ye , Z. Zhu , Z. Fan , Adv. Funct. Mater. 2021, 31, 2102151.

[10] H. L. A. Dickinson , M. D. Symes , Electrochem. Commun. 2022, 135, 107212.

[11] L. Ma , W. Hu , Q. Pan , L. Zou , Z. Zou , K. Wen , H. Yang , J. CO2 Util. 2019, 34, 108.

[12] X. Feng , K. Jiang , S. Fan , M. W. Kanan , J. Am. Chem. Soc. 2015, 137, 4606. 25835085 10.1021/ja5130513

[13] W. Zhu , Y. J. Zhang , H. Zhang , H. Lv , Q. Li , R. Michalsky , A. A. Peterson , S. Sun , J. Am. Chem. Soc. 2014, 136, 16132. 25380393 10.1021/ja5095099

[14] D. R. Kauffman , D. Alfonso , C. Matranga , H. Qian , R. Jin , J. Am. Chem. Soc. 2012, 134, 10237. 22616945 10.1021/ja303259q

[15] M. Wang , K. Torbensen , D. Salvatore , S. Ren , D. Joulié , F. Dumoulin , D. Mendoza , B. Lassalle‐Kaiser , U. Işci , C. P. Berlinguette , M. Robert , Nat. Commun. 2019, 10, 3602. 31399585 10.1038/s41467-019-11542-w PMC6689005

[16] P. Kumar , K. Kannimuthu , A. S. Zeraati , S. Roy , X. Wang , X. Wang , S. Samanta , K. A. Miller , M. Molina , D. Trivedi , J. Abed , M. A. Campos Mata , H. Al‐Mahayni , J. Baltrusaitis , G. Shimizu , Y. A. Wu , A. Seifitokaldani , E. H. Sargent , P. M. Ajayan , J. Hu , M. G. Kibria , J. Am. Chem. Soc. 2023, 145, 8052. 36994816 10.1021/jacs.3c00537

[17] H. T. Chung , D. A. Cullen , D. Higgins , B. T. Sneed , E. F. Holby , K. L. More , P. Zelenay , Science 2017, 357, 479. 28774924 10.1126/science.aan2255

[18] W. Ju , A. Bagger , G. P. Hao , A. S. Varela , I. Sinev , V. Bon , B. Roldan Cuenya , S. Kaskel , J. Rossmeisl , P. Strasser , Nat. Commun. 2017, 8, 944. 29038491 10.1038/s41467-017-01035-z PMC5643516

[19] J. Su , R. Ge , Y. Dong , F. Hao , L. Chen , J. Mater. Chem. A Mater. 2018, 6, 14025.

[20] G. Wang , Y. He , S. Hwang , D. A. Cullen , M. A. Uddin , L. Langhorst , B. Li , S. Karakalos , A. J. Kropf , E. C. Wegener , J. Sokolowski , M. Chen , D. Myers , D. Su , K. L. More , S. Litster , G. Wu , Energy Environ. Sci. 2019, 12, 250.

[21] H. Zhang , S. Hwang , M. Wang , Z. Feng , S. Karakalos , L. Luo , Z. Qiao , X. Xie , C. Wang , D. Su , Y. Shao , G. Wu , J. Am. Chem. Soc. 2017, 139, 14143. 28901758 10.1021/jacs.7b06514

[22] Z. Y. Wu , M. Karamad , X. Yong , Q. Huang , D. A. Cullen , P. Zhu , C. Xia , Q. Xiao , M. Shakouri , F. Y. Chen , J. Y. (Timothy) Kim , Y. Xia , K. Heck , Y. Hu , M. S. Wong , Q. Li , I. Gates , S. Siahrostami , H. Wang , Nat. Commun. 2021, 12, 2870. 34001869 10.1038/s41467-021-23115-x PMC8128876

[23] R. Yang , T. R. Dahn , J. R. Dahn , J. Electrochem. Soc. 2009, 156, B493.

[24] G. C. K. Liu , J. R. Dahn , Appl. Catal. A Gen. 2008, 347, 43.

[25] R. Bashyam , P. Zelenay , Nature 2006, 443, 63. 16957726 10.1038/nature05118

[26] J. Weiss , H. Zhang , P. Zelenay , J. Electroanal. Chem. 2020, 875, 114696.

[27] V. Goellner , C. Baldizzone , A. Schuppert , M. T. Sougrati , K. Mayrhofer , F. Jaouen , Phys. Chem. Chem. Phys. 2014, 16, 18454. 25070913 10.1039/c4cp02882a

[28] Y. Gao , M. Hou , M. Qi , L. He , H. Chen , W. Luo , Z. Shao , Front. Energy 2021, 15, 421.

[29] K. Kumar , L. Dubau , M. Mermoux , J. Li , A. Zitolo , J. Nelayah , F. Jaouen , F. Maillard , Angew. Chem., Int. Ed. 2020, 59, 3235. 10.1002/anie.201912451 31799800

[30] T. N. Nguyen , Z. Chen , A. S. Zeraati , H. S. Shiran , S. M. Sadaf , M. G. Kibria , E. H. Sargent , C. T. Dinh , J. Am. Chem. Soc. 2022, 144, 13254. 35796714 10.1021/jacs.2c04081

[31] A. S. Varela , Curr. Opin. Green Sustain. Chem. 2020, 26, 100371. 10.1016/j.cogsc.2020.100369 PMC7276142 32835134

[32] F. Ismail , A. Abdellah , H. J. Lee , V. Sudheeshkumar , W. Alnoush , D. C. Higgins , ACS Appl. Energy Mater. 2022, 5, 430.

[33] C. Li , W. Ju , S. Vijay , J. Timoshenko , K. Mou , D. A. Cullen , J. Yang , X. Wang , P. Pachfule , S. Brückner , H. S. Jeon , F. T. Haase , S. C. Tsang , C. Rettenmaier , K. Chan , B. R. Cuenya , A. Thomas , P. Strasser , Angew. Chem., Int. Ed. 2022, 61, 202114707. 10.1002/anie.202114707 PMC9306911 35102658

[34] M. Zhang , T. S. Wu , S. Hong , Q. Fan , Y. L. Soo , J. Masa , J. Qiu , Z. Sun , ACS Sustain. Chem. Eng. 2019, 7, 15030.

[35] W. Wang , K. Chen , Y. Sun , S. Zhou , M. Zhang , J. Yuan , Appl. Mater. Today 2022, 29, 101619.

[36] H. B. Yang , S. F. Hung , S. Liu , K. Yuan , S. Miao , L. Zhang , X. Huang , H. Y. Wang , W. Cai , R. Chen , J. Gao , X. Yang , W. Chen , Y. Huang , H. M. Chen , C. M. Li , T. Zhang , B. Liu , Nat. Energy 2018, 3, 140.

[37] Q. Sun , Y. Dong , Z. Wang , S. Yin , C. Zhao , Small 2018, 14, 1704137. 10.1002/smll.201704137 29484816

[38] M. Gong , D. Y. Wang , C. C. Chen , B. J. Hwang , H. Dai , Nano Res. 2016, 9, 28.

[39] Z. Li , X. Wen , F. Chen , Q. Zhang , Q. Zhang , L. Gu , J. Cheng , B. Wu , N. Zheng , ACS Catal. 2021, 11, 8798.

[40] H. Zhang , H. T. Chung , D. A. Cullen , S. Wagner , U. I. Kramm , K. L. More , P. Zelenay , G. Wu , Energy Environ. Sci. 2019, 12, 2548.

[41] J. Tang , R. R. Salunkhe , H. Zhang , V. Malgras , T. Ahamad , S. M. Alshehri , N. Kobayashi , S. Tominaka , Y. Ide , J. H. Kim , Y. Yamauchi , Sci. Rep. 2016, 6, 30295. 27471193 10.1038/srep30295 PMC4965863

[42] C. J. Thambiliyagodage , S. Ulrich , P. T. Araujo , M. G. Bakker , Carbon NY 2018, 134, 452.

[43] S. J. Goldie , S. Jiang , K. S. Coleman , Mater. Adv. 2021, 2, 3353.

[44] M. Sevilla , A. B. Fuertes , Carbon NY 2006, 44, 468.

[45] K. Artyushkova , A. Serov , S. Rojas‐Carbonell , P. Atanassov , J. Phys. Chem. C 2015, 119, 25917.

[46] X. Cui , S. Yang , X. Yan , J. Leng , S. Shuang , P. M. Ajayan , Z. Zhang , Adv. Funct. Mater. 2016, 26, 5708.

[47] L. Li , Y. Wen , G. Han , Y. Liu , Y. Song , W. Zhang , J. Sun , L. Du , F. Kong , Y. Ma , Y. Gao , J. Wang , C. Du , G. Yin , Chem. Eng. J. 2022, 437, 135320.

[48] M. G. Kibria , J. P. Edwards , C. M. Gabardo , C. T. Dinh , A. Seifitokaldani , D. Sinton , E. H. Sargent , Adv. Mater. 2019, 31, 1807166. 10.1002/adma.201807166 31095806

[49] X. Wang , W. Ju , L. Liang , M. Riyaz , A. Bagger , M. Filippi , J. Rossmeisl , P. Strasser , Angew. Chem. 2024, 136, 202401821. 10.1002/anie.202401821 38467562

[50] X. Wang , J. F. de Araújo , W. Ju , A. Bagger , H. Schmies , S. Kühl , J. Rossmeisl , P. Strasser , Nat. Nanotechnol. 2019, 14, 1063. 31591526 10.1038/s41565-019-0551-6

[51] T. Möller , M. Filippi , S. Brückner , W. Ju , P. Strasser , Nat. Commun. 2023, 14, 5680. 37709744 10.1038/s41467-023-41278-7 PMC10502113

[52] M. G. Lee , X. Y. Li , A. Ozden , J. Wicks , P. Ou , Y. Li , R. Dorakhan , J. Lee , H. K. Park , J. W. Yang , B. Chen , J. Abed , R. dos Reis , G. Lee , J. E. Huang , T. Peng , Y. H. (Cathy) Chin , D. Sinton , E. H. Sargent , Nat. Catal. 2023, 6, 310.

[53] J. E. Samad , J. Keels , J. R. Regalbuto , Catal. Lett. 2016, 146, 157.

[54] M. Schreier , J. R. Regalbuto , J. Catal. 2004, 225, 190.

[55] W. A. Spieker , J. R. Regalbuto , Chem. Eng. Sci. 2001, 56, 3491.

[56] J. T. Miller , M. Schreier , A. J. Kropf , J. R. Regalbuto , J. Catal. 2004, 225, 203.

