# Supplementary material for: Atomically Dispersed Ni‐N‐C Catalysts for Electrochemical CO2 Reduction
Source: Small. 2025 Jan 16;21(10):2412162. doi: 10.1002/smll.202412162 (PMC11899493; doi:10.1002/smll.202412162)
Supplement: Supplementary file 1 — Supporting Information [file SMLL-21-2412162-s001.docx]

Supporting Information

**Atomically Dispersed Ni-N-C Catalysts for Electrochemical CO_2_ Reduction**

John Weiss^a*^, Yanghua He^a^, David A. Cullen^b^, Angelica Benavidez^c^, Jeremy D. Jernigen^a^, Hanguang Zhang^a^, Luigi Osmieri^a^, and Piotr Zelenay^a*^

*^a^  Materials Physics and Applications Division, Los Alamos National Laboratory, Los Alamos, NM 87545, USA*

*^b^ Center for Nanophase Materials Science, Oak Ridge National Laboratory, Oak Ridge, TN 37830, USA*

*^c^ Department of Chemical and Biological Engineering, University of New Mexico, Albuquerque, NM 87131, USA*

* Corresponding authors: johnweiss2028@u.northwestern.edu (John Weiss),

zelenay@lanl.gov (Piotr Zelenay)

**S1. Electron energy loss spectroscopy (EELS)**

*
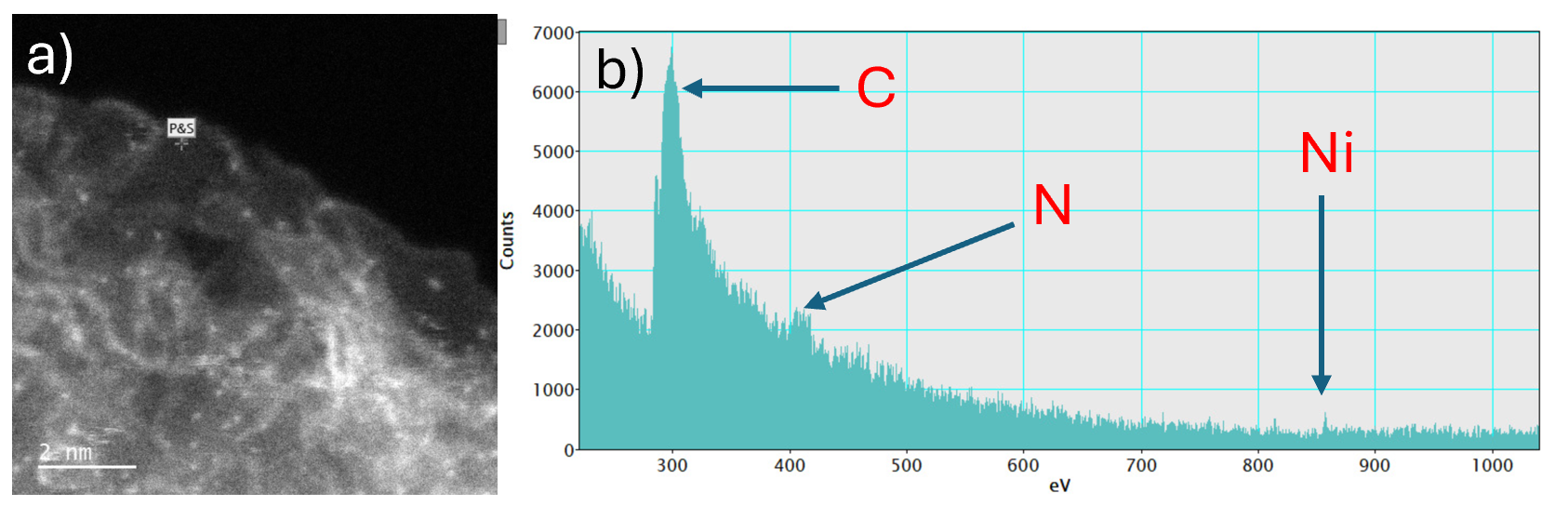
*

***Figure S1****: Ni_0.5%_-N-C catalyst. (****a****) ADF-STEM, and (****b****) Electron energy loss spectroscopy (EELS) of a single nickel atom site coordinated to nitrogen and carbon.*

*
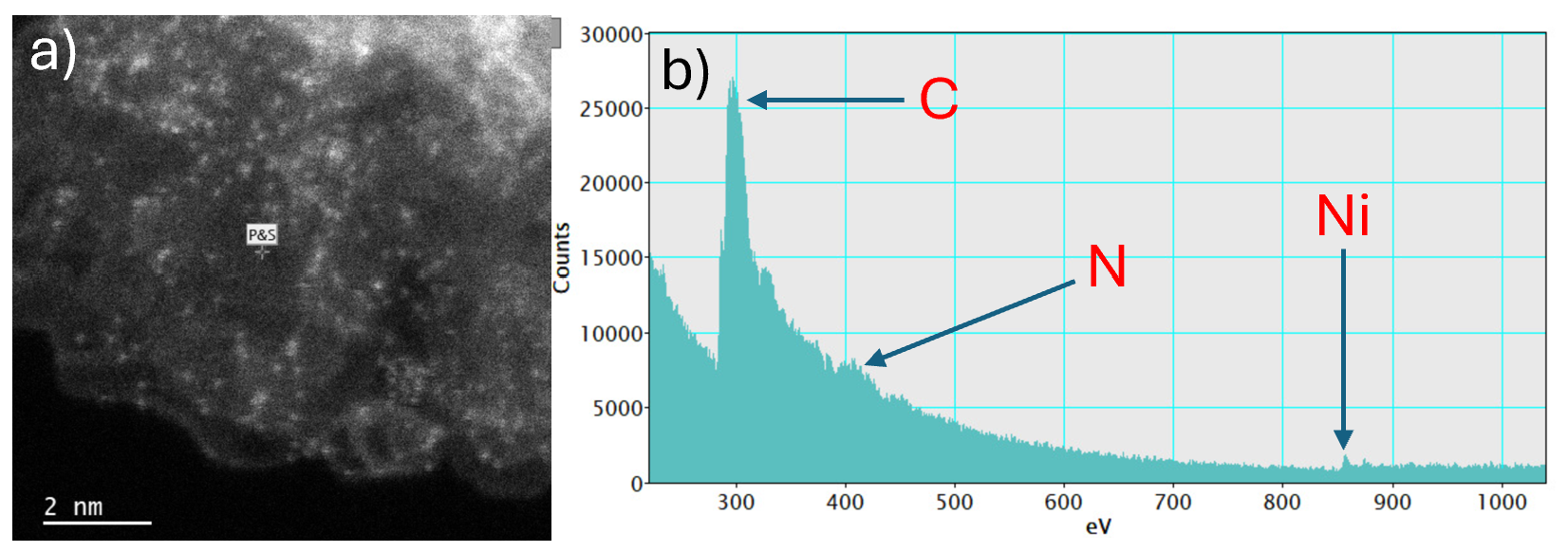
*

***Figure S2****: Ni_1.0%_-N-C catalyst. (****a****) ADF-STEM, and (****b****) Electron energy loss spectroscopy (EELS) of a single nickel atom site coordinated to nitrogen and carbon.*

*
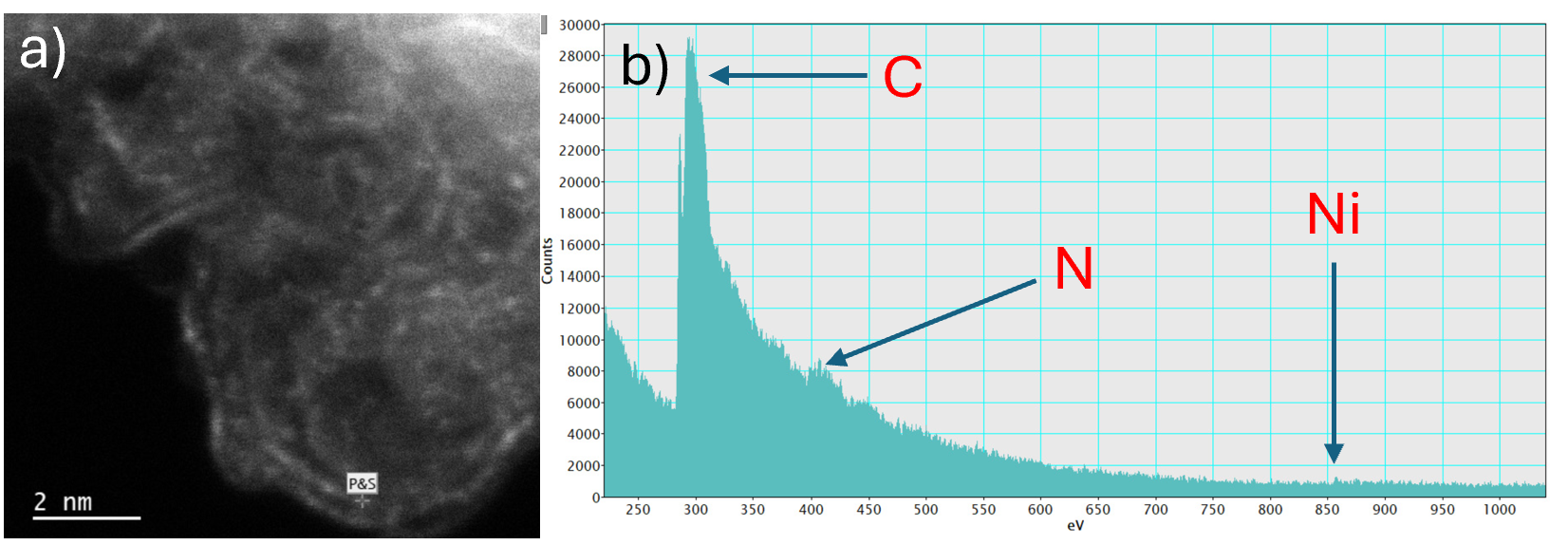
*

***Figure S3****: Ni_1.9%_-N-C catalyst. (****a****) ADF-STEM, and (****b****) Electron energy loss spectroscopy (EELS) of a single nickel atom site coordinated to nitrogen and carbon.*

*
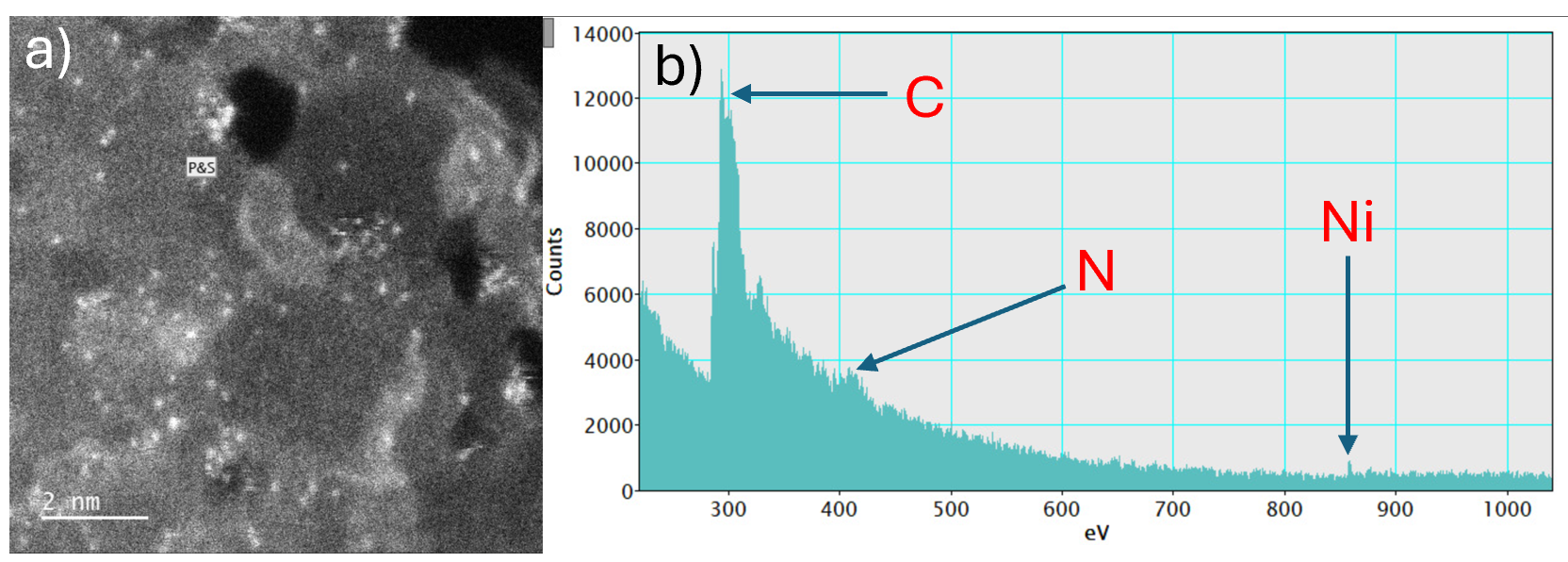
*

***Figure S4****: Ni_4.2%_-N-C catalyst. (****a****) ADF-STEM, and (****b****) electron energy loss spectroscopy (EELS) of a single nickel atom site coordinated to nitrogen and carbon.*

*
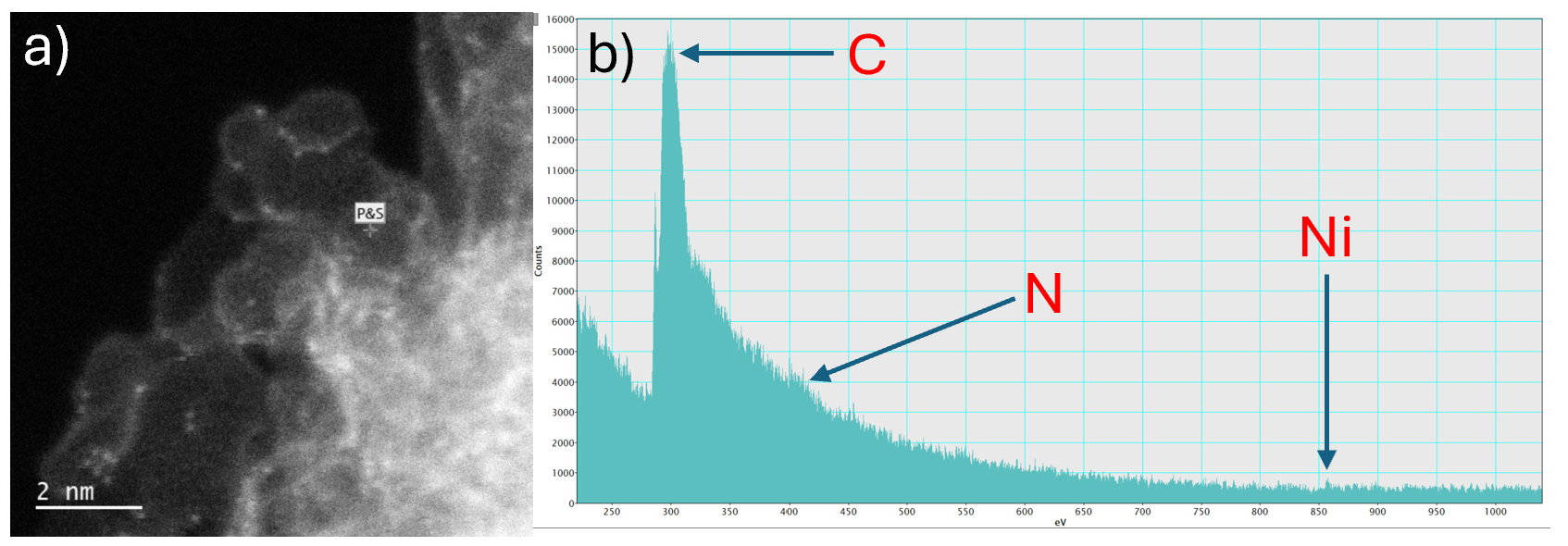
*

***Figure S5****: Ni_6.9%_-N-C catalyst. (****a****) ADF-STEM, and (****b****) Electron energy loss spectroscopy (EELS) of a single nickel atom site coordinated to nitrogen and carbon.*

**S2. Energy-dispersive X-ray spectroscopy (EDS)**


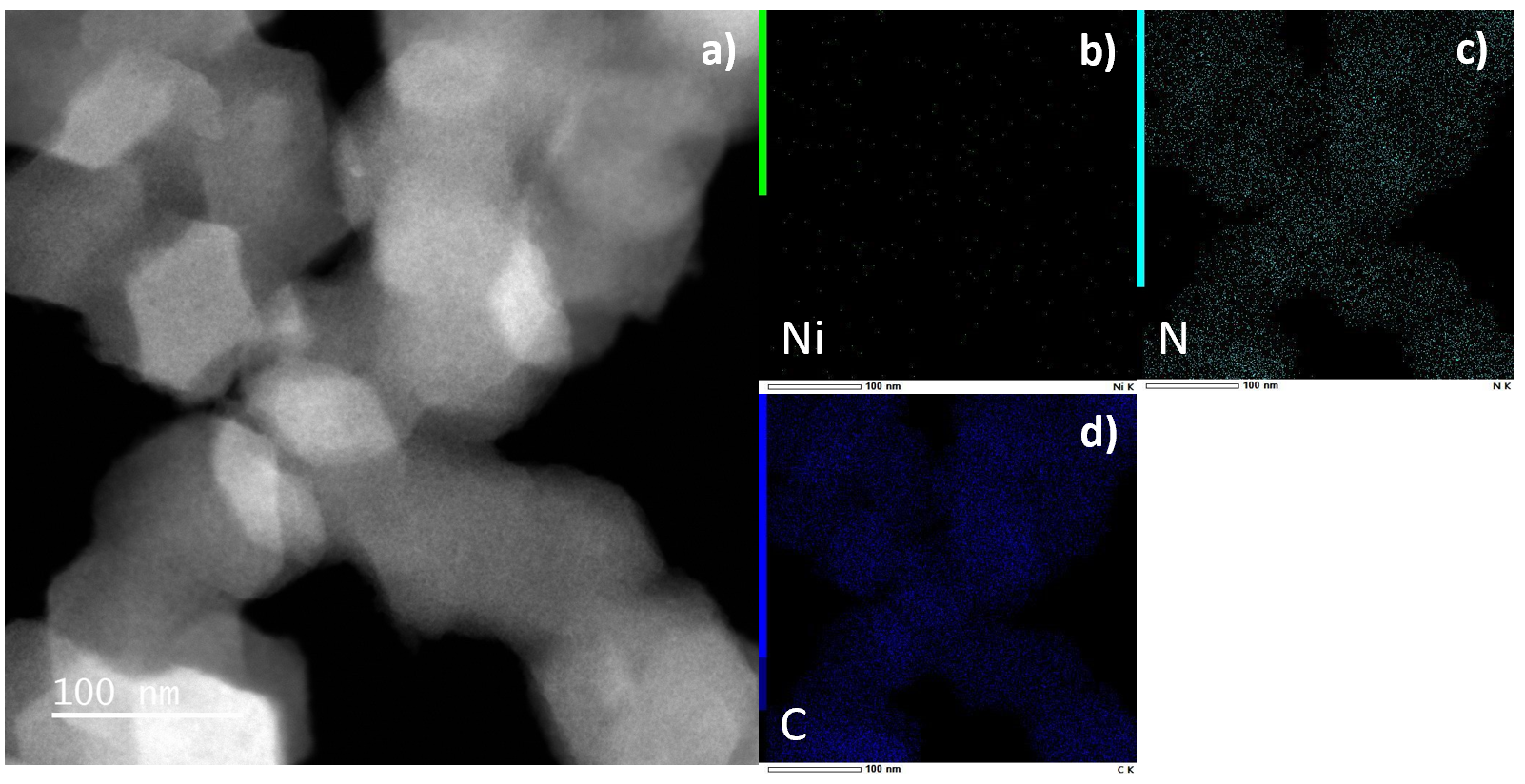


***Figure S6****: (****a****) ADF-STEM image, (****b****) EDS Ni map, (****c****) EDS N map, and (****d****) EDS C map for Ni_0.0%_-N-C.*


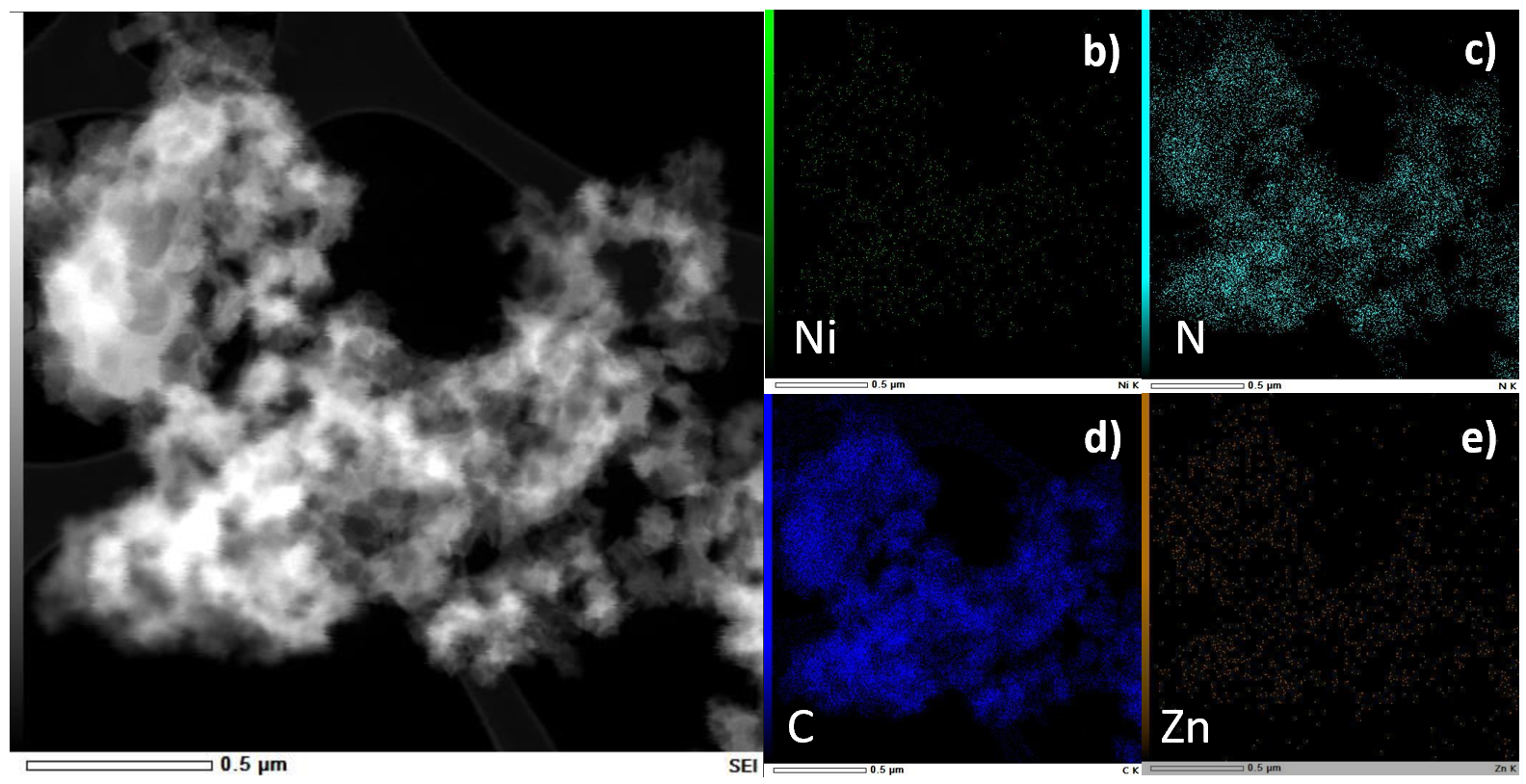


***Figure S7****: (****a****) ADF-STEM image, (****b****) EDS Ni map, (****c****) EDS N map, (****d****) EDS C map, and (****e****) EDS Zn map for Ni_0.5%_-N-C.*


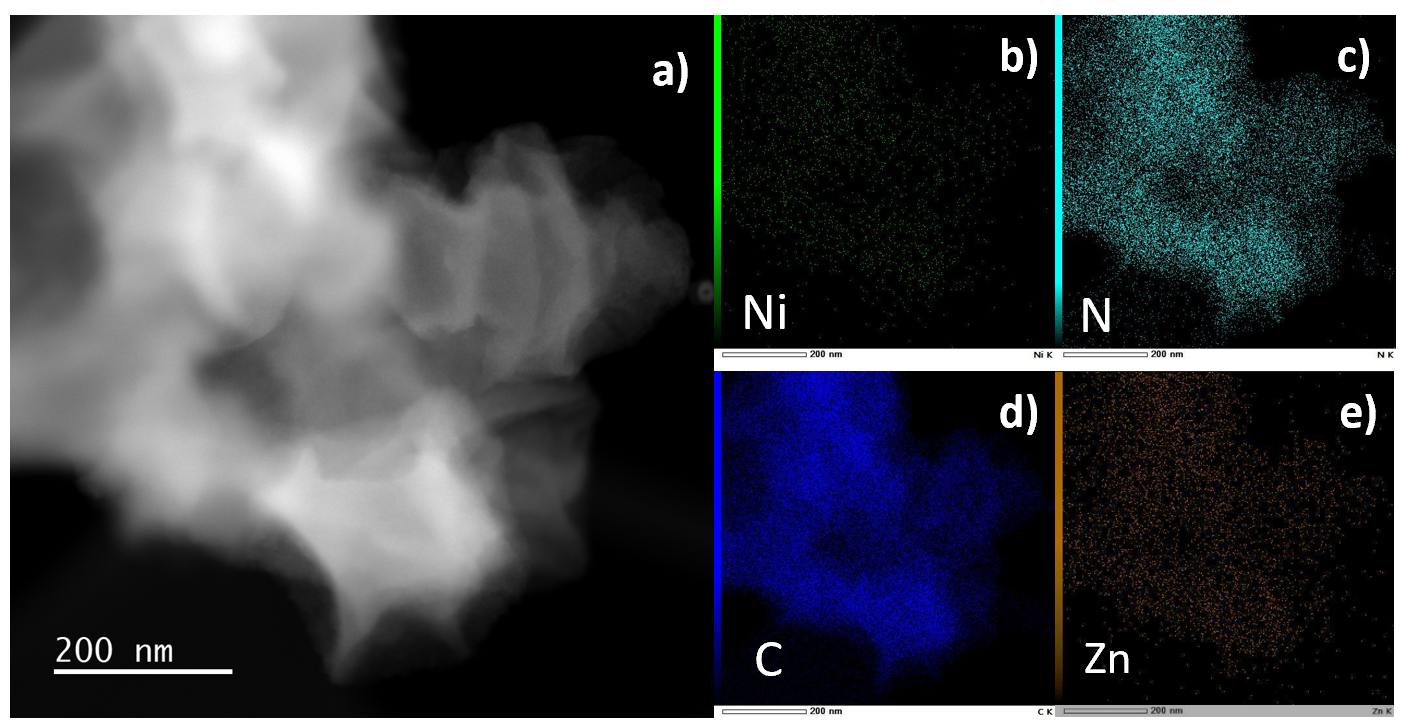


***Figure S8****: (****a****) ADF-STEM map, (****b****) EDS Ni map, (****c****) EDS N map, (****d****) EDS C map, and (****e****) EDS Zn map for Ni_1.0%_-N-C.*


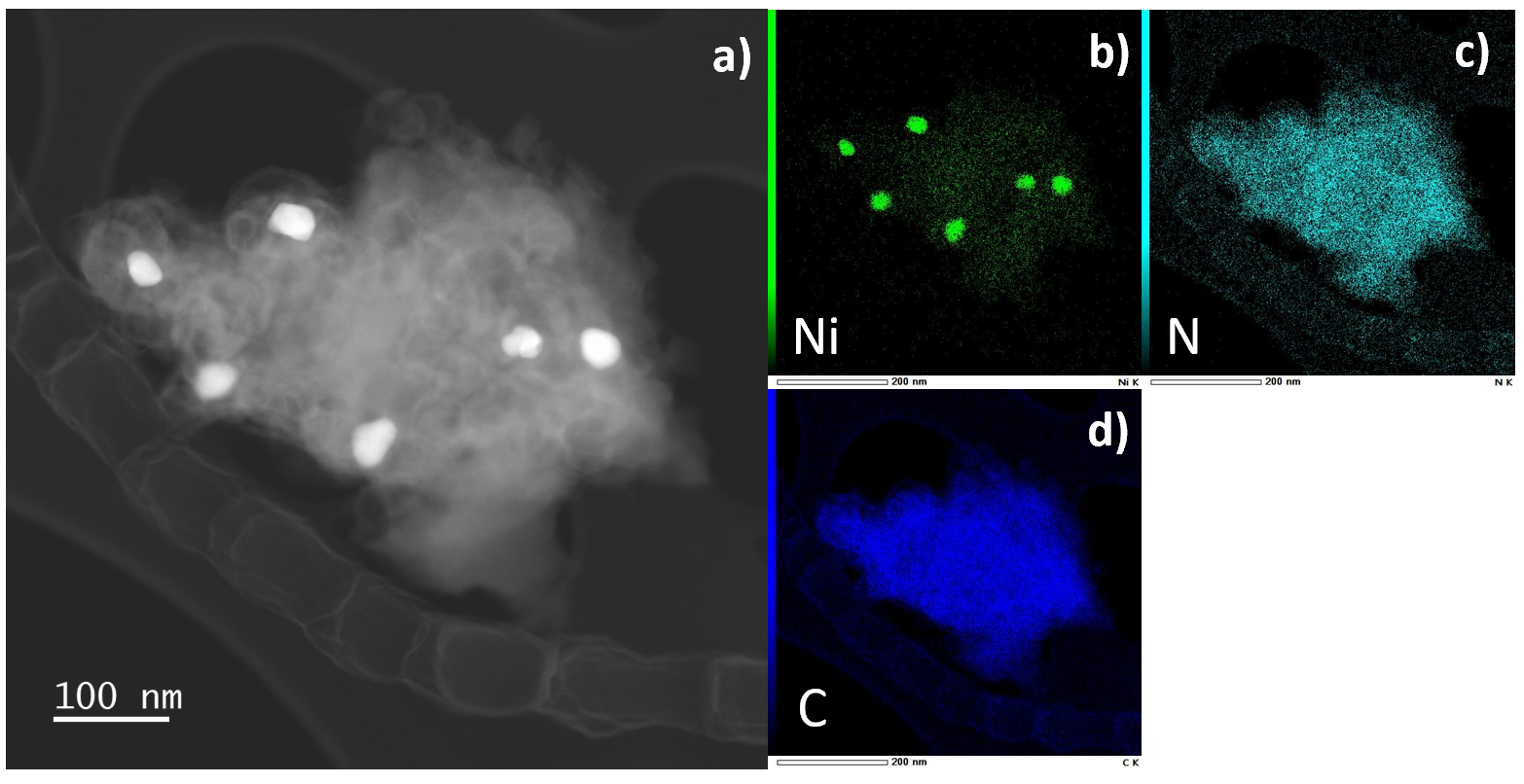


***Figure S9****: (****a****) ADF-STEM image, (****b****) EDS Ni map, (****c****) EDS N map, and (****d****) EDS C map for Ni_1.9%_-N-C.*


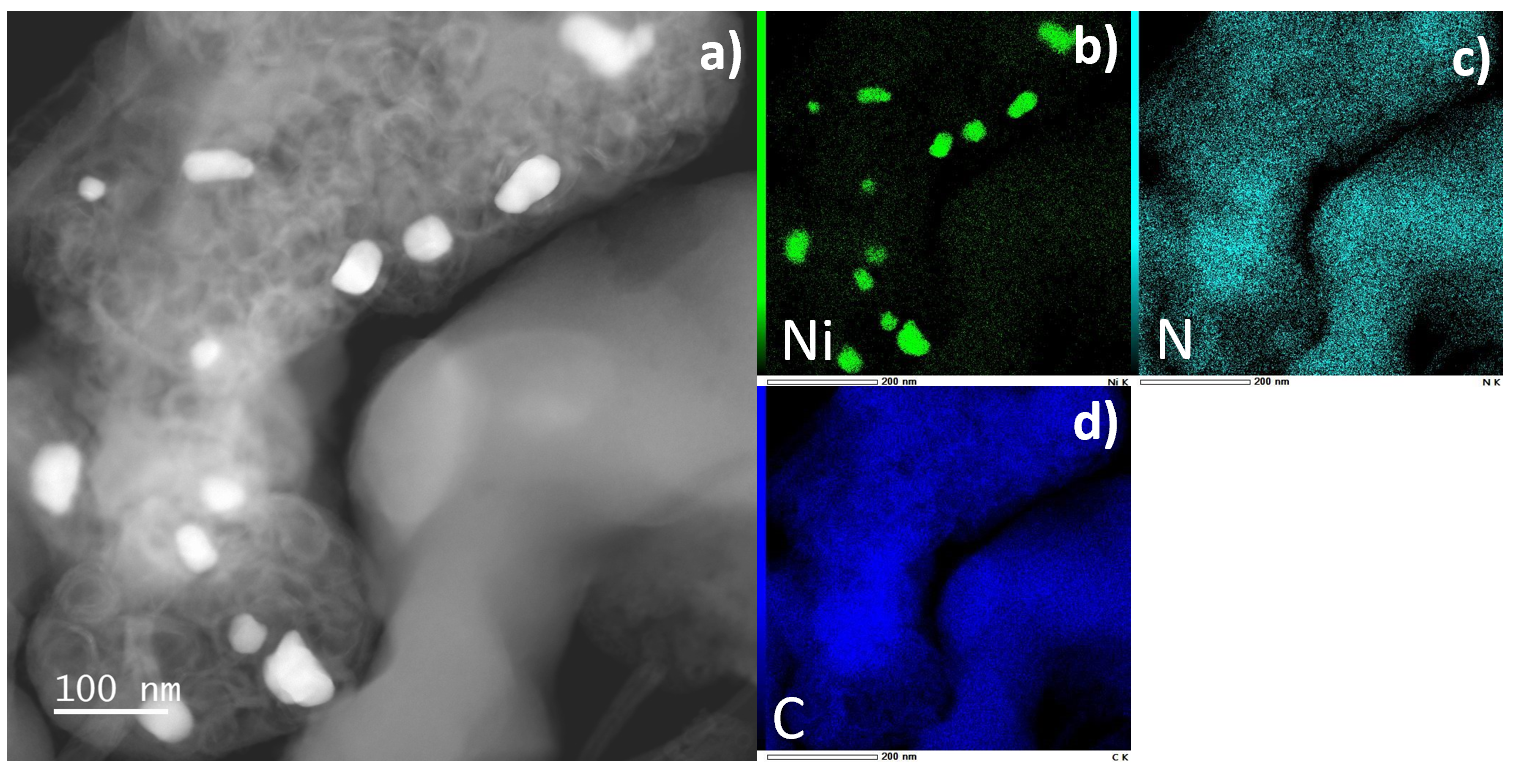


***Figure S10****: (****a****) ADF-STEM image, (****b****) EDS Ni map, (****c****) EDS N map, and (****d****) EDS C map for Ni_4.2%_-N-C.*


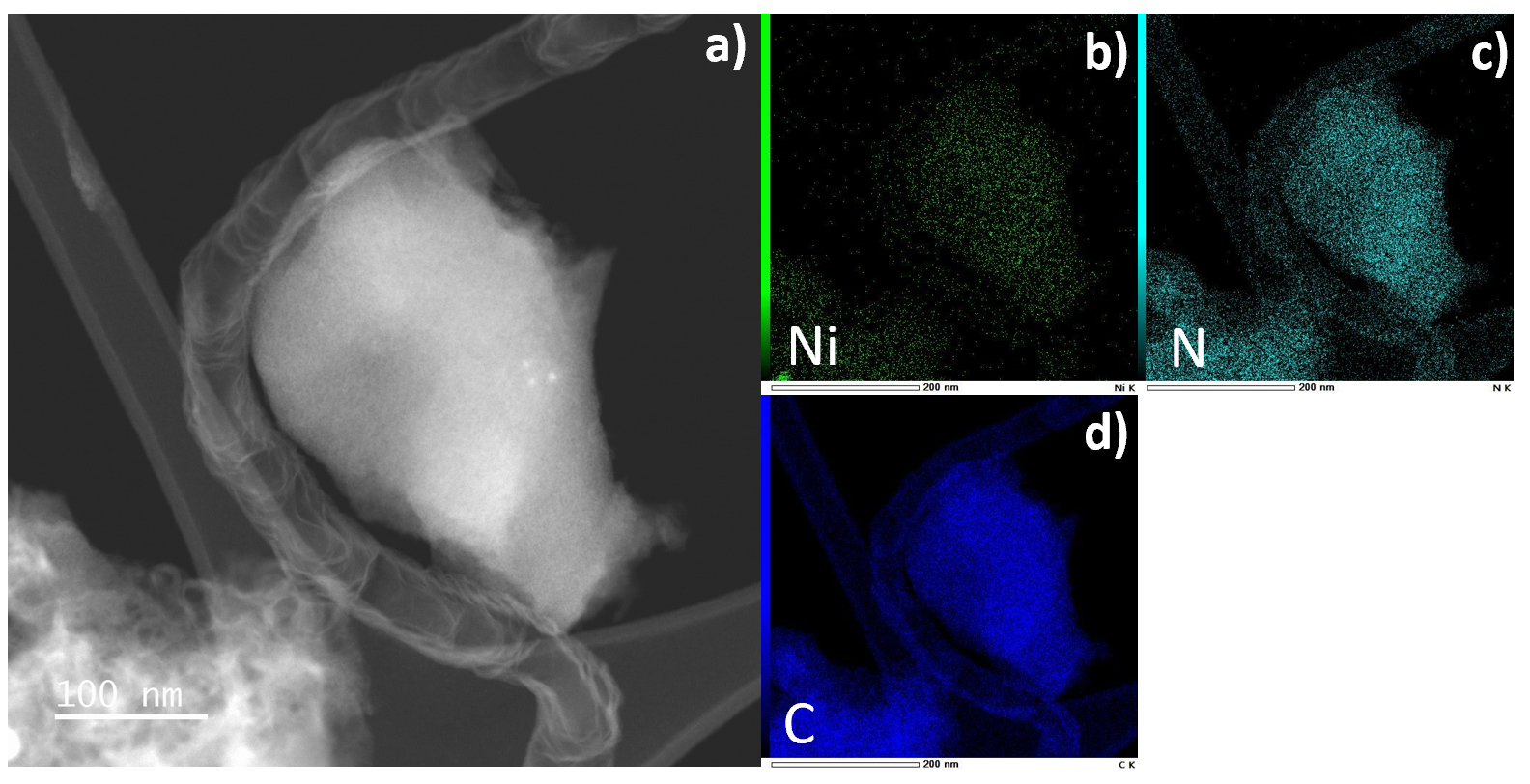


***Figure S11****: (****a****) ADF-STEM image, (****b****) EDS Ni map, (****c****) EDS N map, and (****d****) EDS C map for Ni_6.9%_-N-C.*


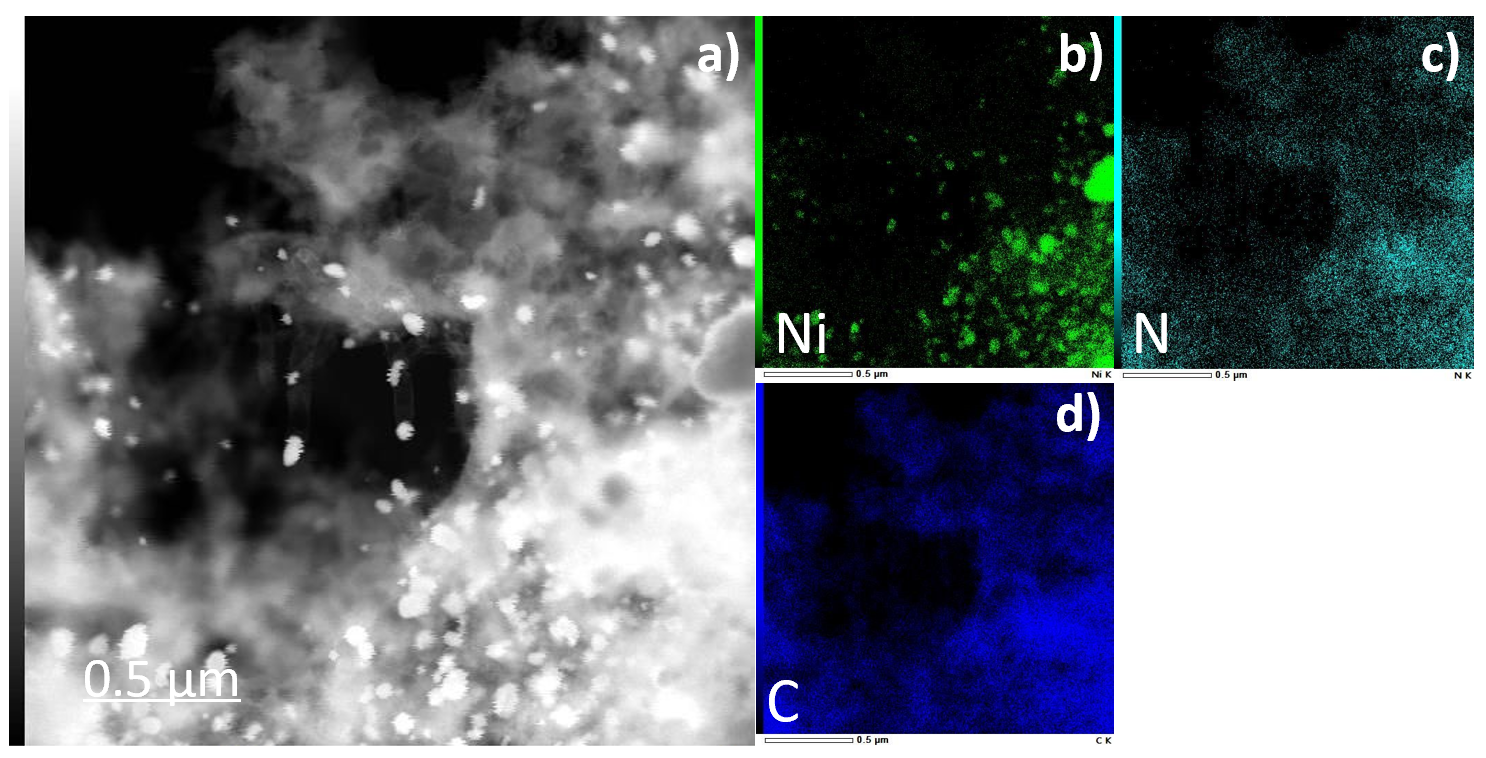


***Figure S12****: (****a****) ADF-STEM image, (****b****) EDS Ni map, (****c****) EDS N map, and (****d****) EDS C map for Ni_10.3%_-N-C.*

**S3. X-ray photoelectron spectroscopy (XPS)**


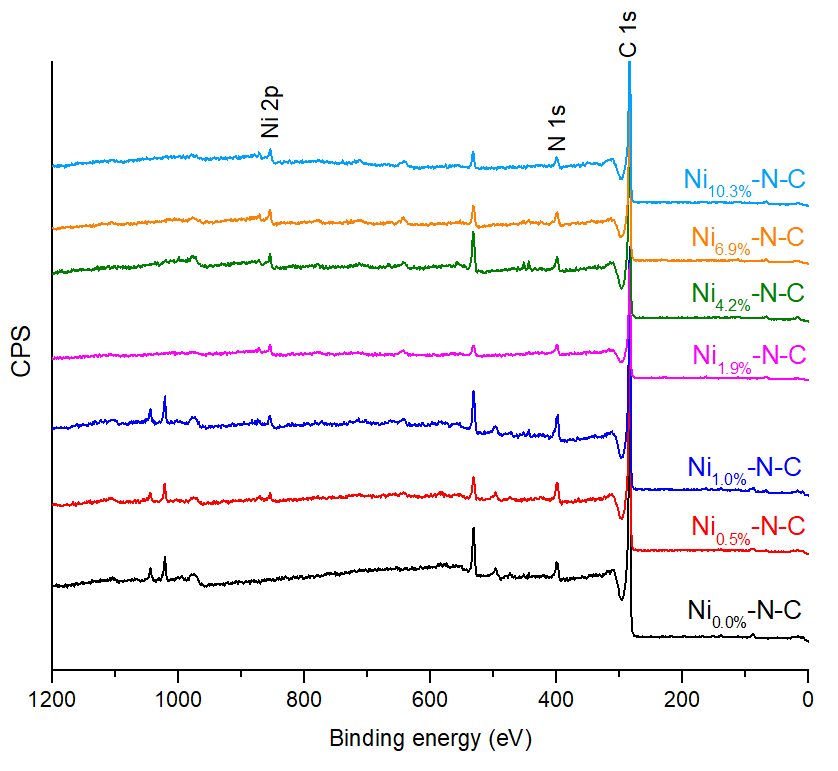


***Figure S13****: XPS survey scans of all Ni-N-C catalysts, with Ni 2p, N 1s, and C 1s regions labeled.*


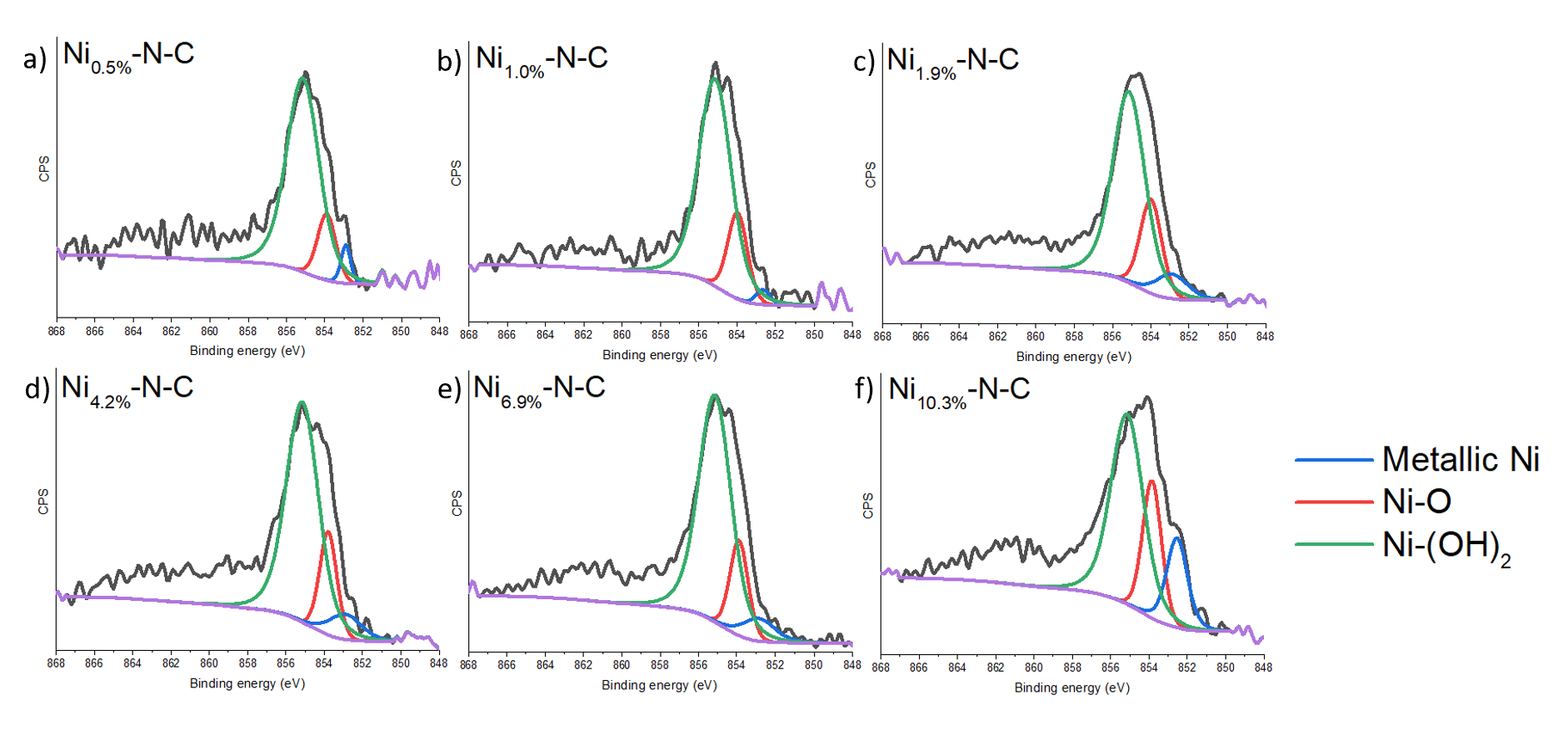


***Figure S14****: XPS Ni 2p spectra of (****a****) Ni_0.5%_-N-C, (****b****) Ni_1.0%_-N-C, (****c****) Ni_1.9%_-N-C, (****d****) Ni_4.2%_-N-C, (****e****) Ni_6.9%_-N-C, (****f****) Ni_10.3%_-N-C.*


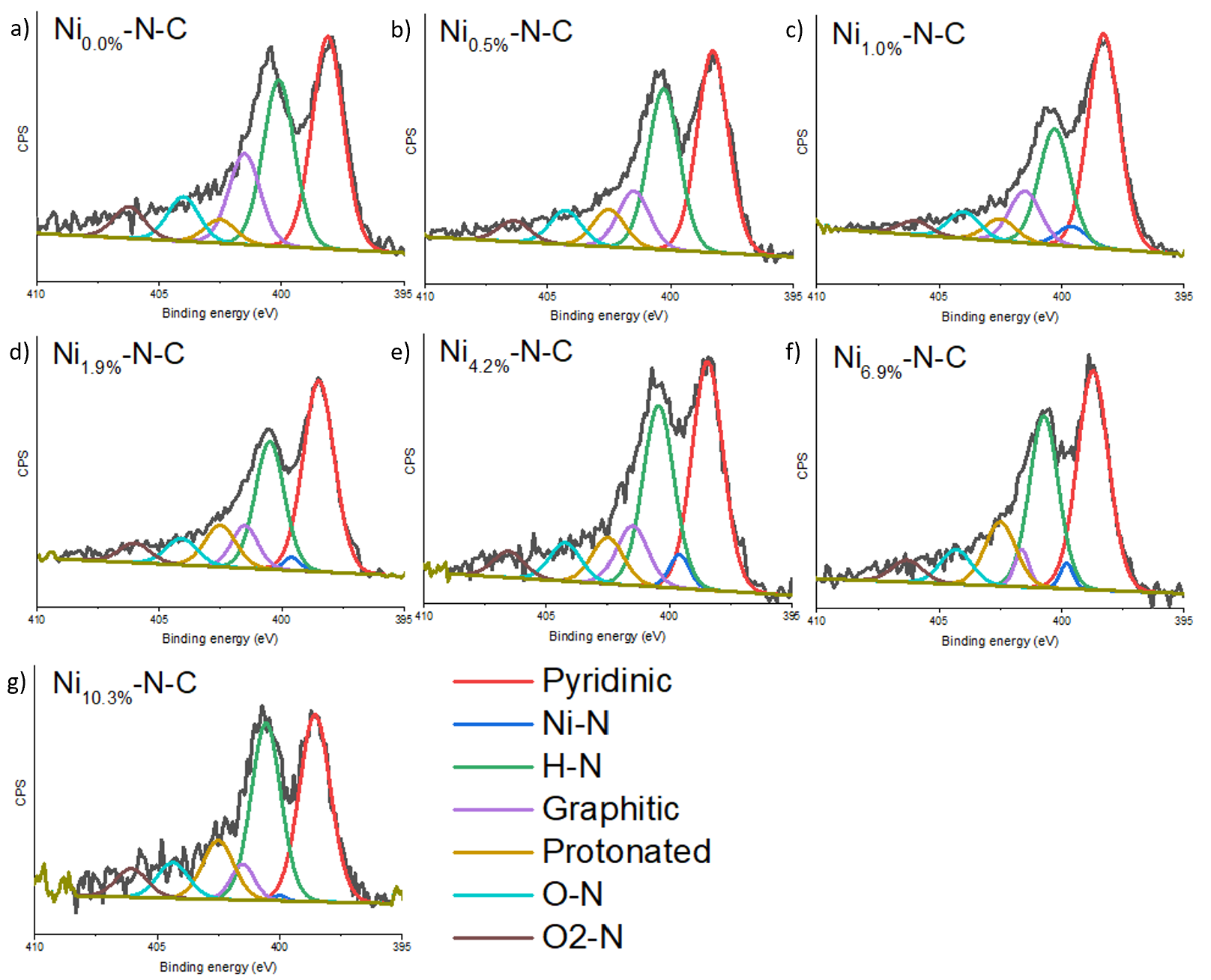


***Figure S15****: XPS N 1s spectra of (****a****) Ni_0.0%_-N-C, (****b****) Ni_0.5%_-N-C, (****c****) Ni_1.0%_-N-C, (****d****) Ni_1.9%_-N-C, (****e****) Ni_4.2%_-N-C, (****f****) Ni_6.9%_-N-C, (****g****) Ni_10.3%_-N-C.*


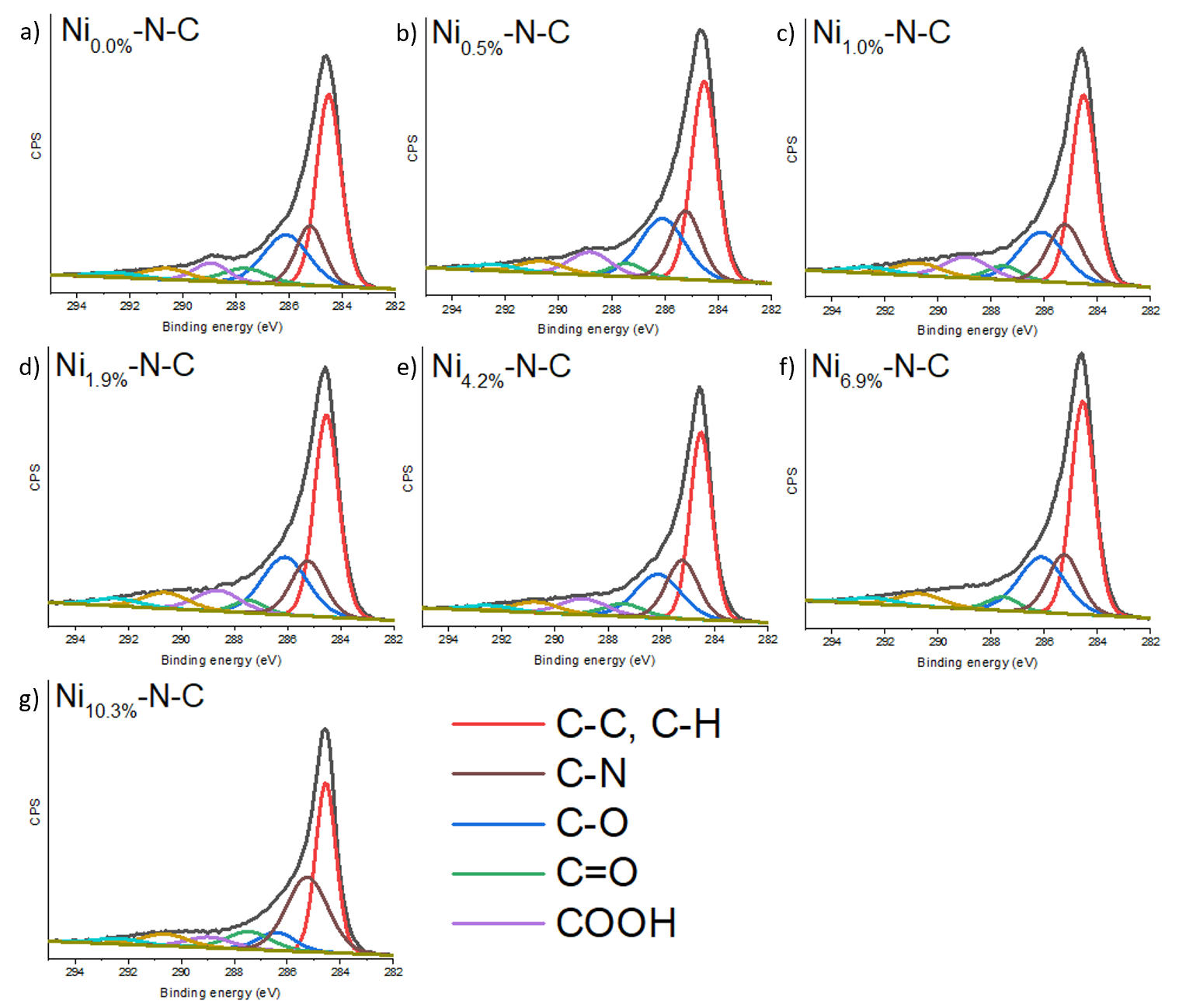


***Figure S16****: XPS C 1s spectra of (****a****) Ni_0.0%_-N-C, (****b****) Ni_0.5%_-N-C, (****c****) Ni_1.0%_-N-C, (****d****) Ni_1.9%_-N-C, (****e****) Ni_4.2%_-N-C, (****f****) Ni_6.9%_-N-C, (****g****) Ni_10.3%_-N-C.*

***Table S1****: Normalized atomic percent of surface species, as determined by XPS survey scans.*

| **Catalyst** | **Ni** | **N** | **C** | **Zn** | **O** |
| --- | --- | --- | --- | --- | --- |
| Ni_0.0%_-N-C | 0.00% | 5.1% | 89% | 0.71% | 5.2% |
| Ni_0.5%_-N-C | 0.68% | 6.2% | 89% | 0.68% | 3.6% |
| Ni_1.0%_-N-C | 0.83% | 7.1% | 86% | 0.82% | 4.9% |
| Ni_1.9%_-N-C | 1.3% | 6.7% | 89% | 0.00% | 3.1% |
| Ni_4.2%_-N-C | 0.94% | 4.5% | 89% | 0.00% | 5.3% |
| Ni_6.9%_-N-C | 1.2% | 5.4% | 89% | 0.00% | 4.3% |
| Ni_10.3%_-N-C | 1.4% | 4.0% | 92% | 0.00% | 2.5% |

***Table S2****: Normalized atomic percent of nickel species, as determined by XPS Ni 2p spectra.*

| **Catalyst** | **Ni metal** | **Ni(OH)_2_** | **NiO** |
| --- | --- | --- | --- |
| Ni_0.5%_-N-C | 5.1% | 79% | 16% |
| Ni_1.0%_-N-C | 3.2% | 79% | 18% |
| Ni_1.9%_-N-C | 7.7% | 71% | 21% |
| Ni_4.2%_-N-C | 8.5% | 72% | 20% |
| Ni_6.9%_-N-C | 7.5% | 76% | 17% |
| Ni_10.3%_-N-C | 19% | 59% | 21% |

***Table S3****: Normalized atomic percent of nitrogen species, as determined by XPS Ni 1s spectra.*

| **Catalyst** | **Pyridinic N** | **Ni-N** | **Hydrated N** | **Graphitized/**  **Protonated N** | **N Oxides** |
| --- | --- | --- | --- | --- | --- |
| Ni_0.0%_-N-C | 38% | 0.00% | 30% | 19% | 12% |
| Ni_0.5%_-N-C | 40% | 0.51% | 31% | 18% | 11% |
| Ni_1.0%_-N-C | 47% | 2.5% | 26% | 16% | 8.8% |
| Ni_1.9%_-N-C | 43% | 1.1% | 28% | 17% | 11% |
| Ni_4.2%_-N-C | 39% | 2.5% | 29% | 18% | 12% |
| Ni_6.9%_-N-C | 40% | 2.0% | 32% | 15% | 11% |
| Ni_10.3%_-N-C | 36% | 0.71% | 34% | 15% | 14% |

***Table S4****: Normalized atomic percent of carbon species, as determined by XPS C 1s spectra.*

| **Catalyst** | **C-C / C-H** | **C-N** | **C-O** | **C=O** | **COOH** |
| --- | --- | --- | --- | --- | --- |
| Ni_0.0%_-N-C | 48% | 18% | 22% | 4.5% | 8.1% |
| Ni_0.5%_-N-C | 44% | 21% | 21% | 5.5% | 8.7% |
| Ni_1.0%_-N-C | 46% | 19% | 23% | 3.9% | 8.4% |
| Ni_1.9%_-N-C | 46% | 18% | 23% | 4.2% | 8.3% |
| Ni_4.2%_-N-C | 44% | 21% | 22% | 5.3% | 8.1% |
| Ni_6.9%_-N-C | 46% | 18% | 22% | 4.2% | 8.5% |
| Ni_10.3%_-N-C | 42% | 37% | 7.5% | 7.4% | 6.8% |

**S4. Rotating disk electrode (RDE) measurements**


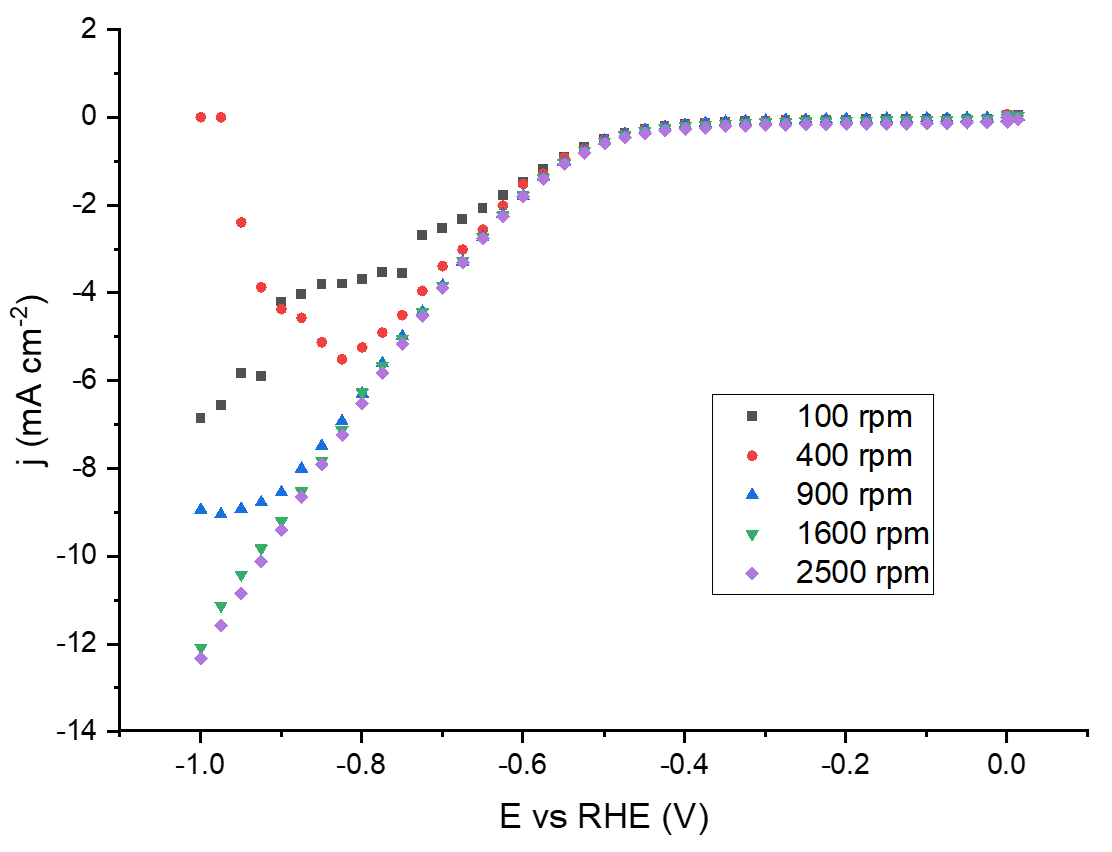


***Figure S17****: Staircase voltammogram for Ni_1.0%_-N-C comparing different rotation speeds at the working electrode.*


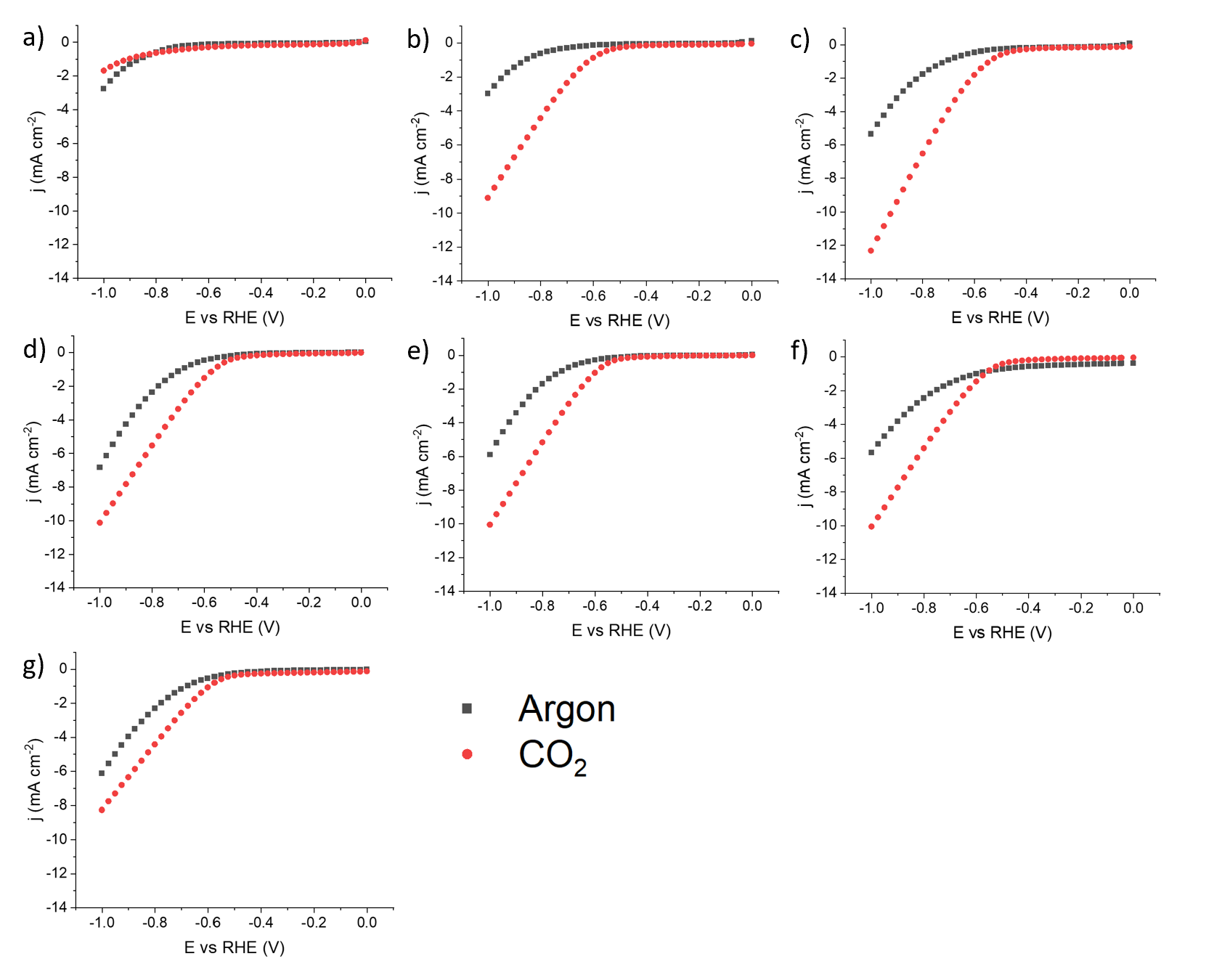


***Figure S18****: Staircase voltammograms taken in both CO_2_- and Ar-saturated 0.1 M KHCO_3_ electrolyte for (****a****) Ni_0.0%_-N-C, (****b****) Ni_0.5%_-N-C, (****c****) Ni_1.0%_-N-C, (****d****) Ni_1.9%_-N-C, (****e****) Ni_4.2%_-N-C, (****f****) Ni_6.9%_-N-C, (****g****) Ni_10.3%_-N-C.*

***Table S5****: Δj (mA cm^-2^) between CO_2_- and Ar-saturated 0.1 M KHCO_3_ electrolyte.*

| **Potential**  **vs. RHE (V)** | **Δj (mA cm^-2^)** | | | | | | |
| --- | --- | --- | --- | --- | --- | --- | --- |
|  | Ni_0.0%_-N-C | Ni_0.5%_-N-C | Ni_1.0%_-N-C | Ni_1.9%_-N-C | Ni_4.2%_-N-C | Ni_6.9%_-N-C | Ni_10.3%_-N-C |
| -0.80 | -0.04 | -3.8 | -4.8 | -3.17 | -3.5 | -3.0 | -2.5 |
| -0.85 | 0.14 | -4.6 | -5.5 | -3.45 | -3.9 | -3.5 | -2.7 |
| -0.90 | 0.35 | -5.3 | -6.2 | -3.56 | -4.2 | -3.9 | -2.8 |
